# Maize stover burning exposure accountable for remarkable environmental and health risk in broiler chickens

**DOI:** 10.1186/s12917-025-04476-7

**Published:** 2025-03-25

**Authors:** Manal A. M. Mahmoud, Abd El-Aziz A. Said, Hanan H. Abd-Elhafeez, Soha A. Soliman, Usama T. Mahmoud

**Affiliations:** 1 Department of Animal Hygiene, Faculty of Veterinary Medicine, Assiut University, Assiut, 71526 Egypt; 2 Chemistry Department, Faculty of Science, Assiut University, Assiut, 71526 Egypt; 3 Department of Cell and Tissues, Faculty of Veterinary Medicine, Assiut University, Assiut, 71526 Egypt; 4 Department of Histology, Faculty of Veterinary Medicine, South Valley University, Qena, 83523 Egypt; 5 Department of Animal, Poultry and Aquatic Life Behaviour and Management, Faculty of Veterinary Medicine, Assiut University, Assiut, 71526 Egypt

**Keywords:** Biomass burning, Haze formation, Hemoconcentration, Elevated liver enzymes, Metaplasia

## Abstract

**Background:**

Biomass burning presents significant environmental and health problems worldwide. Health effects on broilers (as an animal model) exposed to intensive maize stover burning (MSB) were studied. Carbon monoxide (CO) and fine particulate matter (PM2.5) were estimated during the MSB season. Sixty apparently healthy broilers from 12 farms were included for blood-gas analysis, bilirubin, and liver enzyme analysis. In addition, histopathological changes of the lung, liver, and heart were investigated.

**Results:**

Highly significant differences for CO and PM2.5 levels, hemoglobin (Hb), and hematocrit (Hct) values during MSB season were found compared to burning free events which resulted in higher incidence of blood coagulation and cardiovascular diseases risk. Highly significant elevations of liver enzymes were verified during MSB. Respiratory function was significantly decreased due to airway obstruction accompanied by severe tissue damage including pulmonary fibrosis (39%) and metaplasia. Pulmonary and hepatic blood vessel embolisms were indicative of systemic embolic phenomena.

**Conclusion:**

The study highlighted the substantial health risk and a threat to air quality from one season exposure to leftover straw burning. Agriculture waste burning should be banned by legislation to restore the environment and protect health.

**Supplementary Information:**

The online version contains supplementary material available at 10.1186/s12917-025-04476-7.

## Background

Open biomass burning (bushfires, planned to burn, and agriculture waste burning) is a worldwide environmental and health problem which extends in vegetation and urban areas. The United States of America is the third-largest emitter of greenhouse gases from agricultural burning worldwide, after China and India. The 2020 U.S. National Emissions Inventory states that 67,309.81 tons, or around 20% of all PM2.5 emissions, came from burning in agricultural fields [1]. In many developing agricultural countries like Egypt, burning of straw is a common practice for eliminating agricultural waste. The vast expansion of the Egyptian economy resulted in a large annual production of agriculture waste that was left untreated. Total agricultural waste was estimated to be about 35 million tons/year. Only about 31% of this waste is utilized for animal feed or organic fertilizer, and the rest was subjected to open-air burning after crop harvesting [2]. Assiut ranks second and sixth to produce sorghum (977.5 thousand heml) and maize (1669 thousand heml), representing about 27.6% and 7.4% of Egypt’s production, respectively [3].

A cloud of thick smoke blankets Assiut in Upper Egypt during October and November due to intensive burning of maize, after harvesting. The same phenomenon occurs in greater Cairo from the burning of rice straw, resulting in high levels of air pollution and serious environmental and health problems [4]. Burning of agricultural crop residue emits several toxic gases including carbon monoxide (CO), volatile organic compounds, and fine particulate matter (PM) [5]. CO is a common air pollutant generated from burning maize [6]. Many cases of asphyxiation resulting from the “black cloud” which appeared over the city and adjacent villages have been reported in Assiut. Moreover, urban areas far from burning centers are consistently affected by severe air pollution from the smoke which can travel over long distances [7]. Additionally, automobile accidents have been reported in the area during burning episodes due to poor visibility [8]. Farmers are aware of the harmful effects of burning, and satellites are used to spot open burning processes; however, farmers tend to start burning at night to avoid penalties assessed to violators [9]. Although the level of black cloud generated by rice straw burning has decreased in Lower Egypt (Delta and Cairo) compared to 1999, the level of pollution in Upper Egypt is still a dramatic problem. In 2018, the Ministry of the Environment announced changes regarding the waste management system, particularly in dealing with excess leftover straw by providing agricultural shredders to farmers and offering money to encourage them to collect and give the straw to the government instead of burning it [9]. However, the leftover straw exceeded the government’s capacity to manage it in Upper Egypt. In particular, farmers with little amounts of remaining straw, found it easier and cheaper to burn it rather than collecting and transporting it to the main shredder areas [10].

Sever air pollution from open burning is strongly linked to mortality, cardiovascular illness, asphyxiation, and hospital admissions [11, 12]. Symptoms due to smoke inhalation are related to the extent and route of exposure, the level of pollutants, and preexisting health status. Respiratory diseases such as asthma, chronic bronchitis, obstructive pulmonary disease (COPD), exacerbation of cystic fibrosis, and lung cancer are common consequences of particle inhalation [13].

Chickens have been used recently as an excellent model for toxicological, reproductive, inflammatory, and pain related behavior studies. Chickens are inexpensive to purchase, sensitive, easily maintained and show a prompt response to environmental pollutants that make them an invaluable model for toxicological studies [14]. Broilers, a chicken breed with a short lifespan, were used as animal model for health risk assessment in this study. Environmental problems due to agriculture waste burning have been studied in China, Indonesia, and Thailand where crop straw open burning is a common practice [13, 15, 16]. However, health risk assessments due to agriculture waste burning are lacking. Therefore, the purpose of this study was to assess the environmental and health risks associated with agriculture waste burning exposure, which occurs with the annual burning of leftover straw crops in Egypt.

## Results

**Fig. 1:**
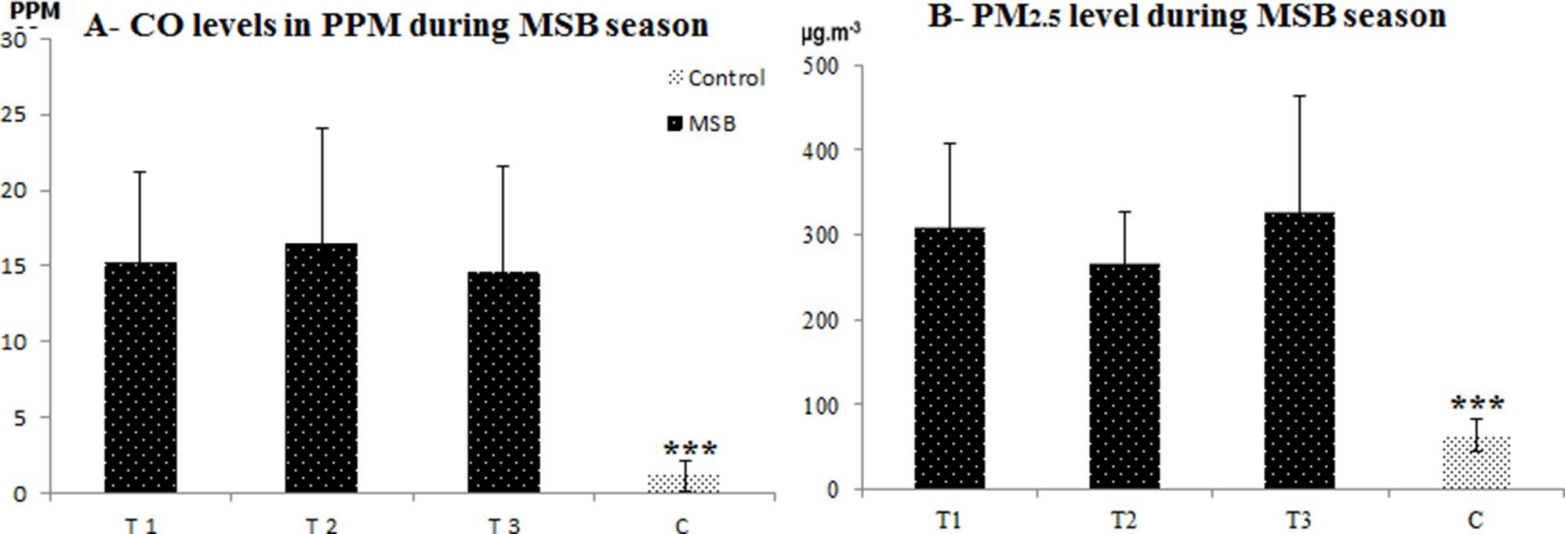
(**A** & **B**): The average Co and PM concentrations during Maize Stover burning (MSB) season and free burning event from six villages in Assiut, Egypt. T1, T2 and T3 were three times sets for Co and PM samples representing the beginning, the middle and the end of MSB season, respectively. C: control samples. * Significant difference *P* < 0.05, ** highly significant difference *P* < 0.01, *** highly significant difference *P* < 0.001

**Fig. 2:**
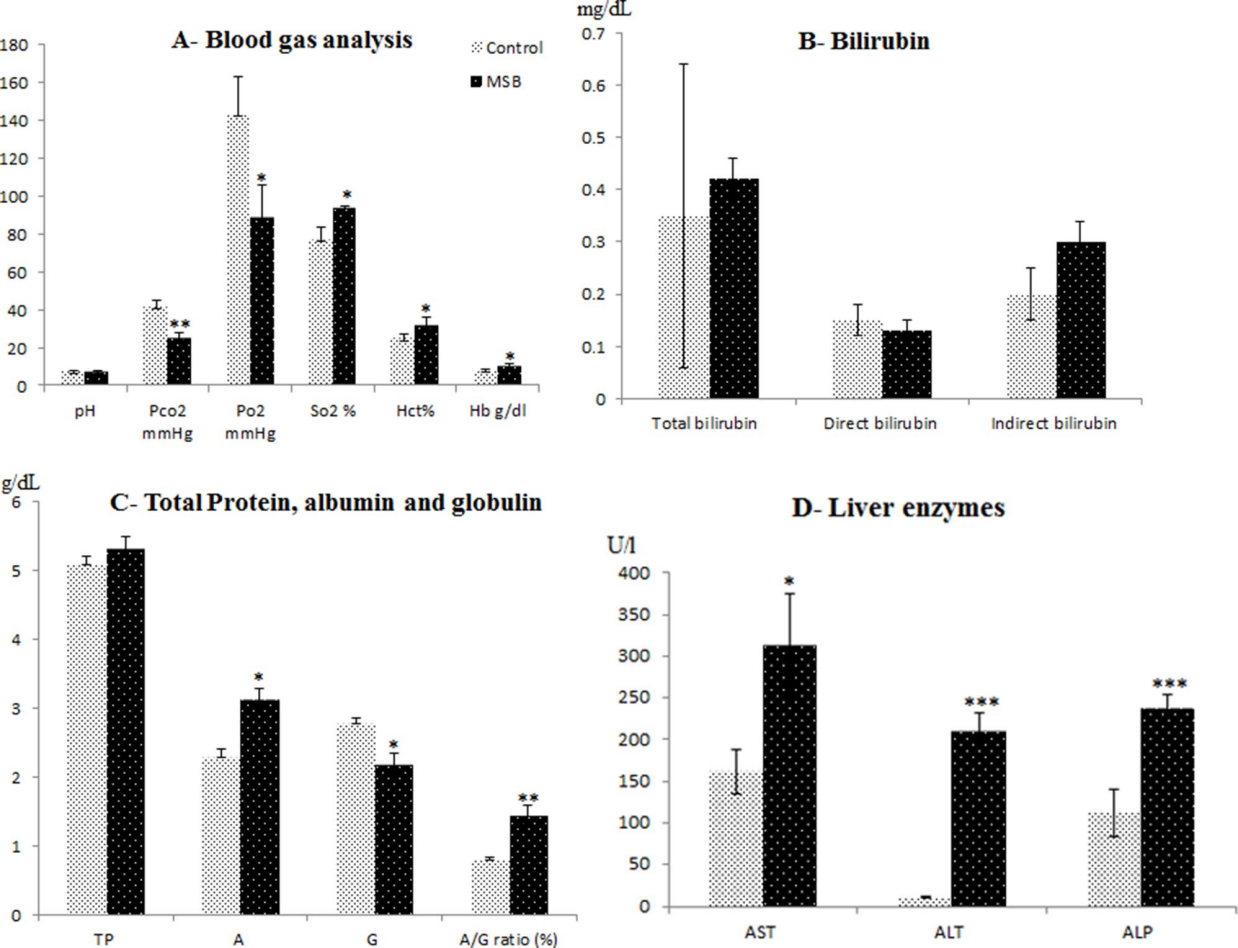
(**A**, **B**, **C** & **D**): Blood gas analysis, bilirubin (TB), total protein (TP), liver enzymes (AST, ALP and ALP) from exposed broilers to Maize Stover burning (MSB) and free burning events. * Significant difference *P* < 0.05, ** highly significant difference *P* < 0.01, *** highly significant difference *P* < 0.001

**Fig. 3:**
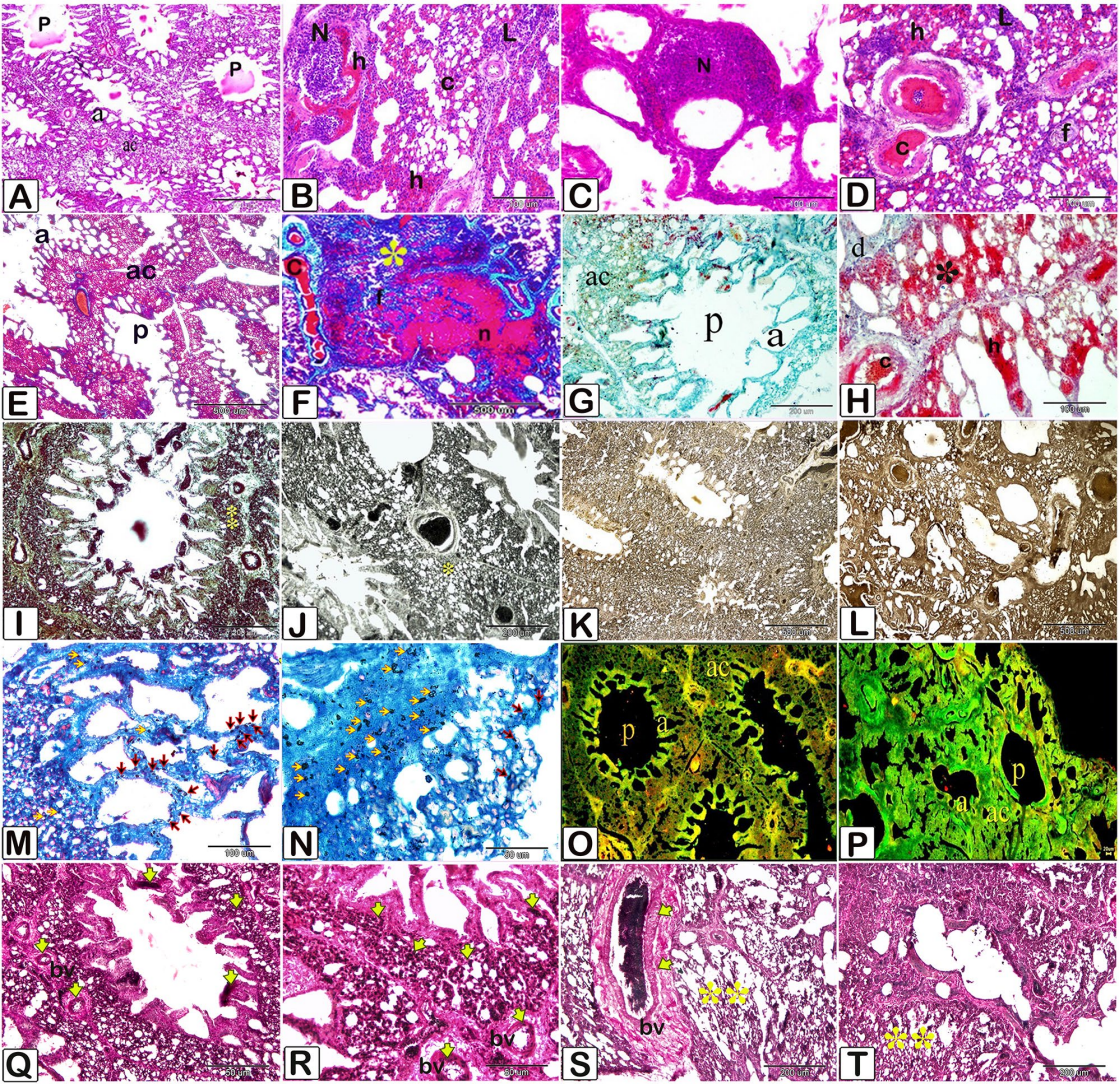
Lung Histopathology including photomicrograph of Hematoxylin and Eosin (**A**-**D**), Crossmon’s trichrome (**E**, **F**), Ziehl-Nelsen (**G**, **H**), Mercury bromophenol blue (**I**, **J**), Gomori calcium method (**K**, **L**), Prussian blue (**M**, **N**), Acridine orange (**O**, **P**), (**Q**, **R**, **S**, **T**), Wiegert’s stain stained paraffin sections of lung. **A**: lung of the control group. The atria (**a**) opened into the parabronchus (**P**). The air capillaries (ac) continued with the atrium. **B**, **C**, **D**: lung of the MSB group. **B**, **C**: nodular (**N**), and diffuse (**L**) mononuclear inflammatory cell infiltrates, Hemorrhage (**h**), congested blood capillaries (**C**). **D**: hyalinized muscle fibers (asterisk), Hemorrhage (**h**), diffuse (**L**) mononuclear inflammatory cell infiltrates, Pulmonary fibrosis (**f**). Congestion (**c**). **E**: Lung of the control group showing the atria (**a**) opened into the parabronchus. The air capillaries (**ac**) continued with the atrium. F: lung of the MSB group. **F**: parabronchus (**P**) loss the architecture that had no atria. Pulmonary fibrosis (asterisks), Vascular congestion (**C**), Necrotic mass (**n**) surrounded by fibrous tissue (**f**). **G**: Lung of the control group showing the atria (**a**) opened into the parabronchus. The air capillaries (**ac**) continued with the atrium. Note parabronchus (**P**), atria (**a**), air capillaries (**ac**). **H**: lung of the MSB group. lipofuscin pigment (*) positive interstitial cells, Hemorrhage (**h**), vascular congestion (**C**), diffuse (**d**) inflammatory cell infiltrates. **I**: lung sample of the control group. Blue coloration indicated positive protein inclusions. Lung tissue including the pneumocytes had a high (**) affinity for mercury bromophenol blue that indicate cellular inclusions of protein nature that was uniformly distributed. **J**: lung sample of the MSB group. Pnuemocytes had a low affinity (*) for bromophenol blue that was indicated by a marked decrease in protein inclusions. Considerable loss and uneven distribution of protein inclusions are detected. **K**: Lung sample of the control group. Lung tissue had a low affinity for alkaline phosphatase. **L**: lung sample of the MSB group had a high affinity for alkaline phosphatase. **M** and **N**: Prussian blue method-stained paraffin sections of the lung for detection of hemosiderin pigments. N: lung sample of the MSB group with a low number of macrophages with hemosiderin pigment (black arrows) and abundant pigment within the interstitial tissue (yellow arrows) in comparison to lung sample of the control group (**M**). Hemosiderin pigments inside the macrophage cells (black arrows) and few pigments within the interstitial tissue (yellow arrows). **O**, **P**: Acridine orange method-stained paraffin sections of the lung for detection of lysosomes. **O**: Lung sample of the control group. Note parabronchus (**P**), atria (**a**), air capillaries (**ac**). Note numerous activated lung macrophages that indicated by the yellow and red reaction. **P**: lung sample of the MSB groups. Less yellowish reaction indicated dismissing the lung macrophages activities. **S** and **T**: lung sample of the MSB group showing reduced elastic fibers interstitial tissue and around the air capillaries (*) and in-wall of a blood vessel (BV, yellow arrow) in comparison to control group (**Q**, **R**)

CO levels during MSB season ranged from 8.7 to 33.2 ppm. There was a highly significant difference between CO levels during MSB season and control samples (Fig. 1). Fine PM_2.5_ was significantly higher during the MSB season than the control. The highest PM_2.5_ level was 611 µg/m^–3^ and the lowest was 193 µg/m^–3^ during MSB season. During the control period the PM_2.5_ concentrations were as low as 64 ± 19 (Fig. 2B). The effects of maize burn exposure on pH and blood gases are noted in Fig. 3A. The broilers’ pH was not affected by MSB and pO_2_ was decreased compared to controls. However, levels of pCO_2_ and HCO_3,_ Hb and Hct were significantly increased in MSB broilers compared to control.

The broilers biochemical parameters are shown in Fig. 3B, C, D. TB (direct and indirect) and TP (Fig.3B, C) did not differ significantly between the MSB and control groups. However, there was a significant decrease in G and increase in A in the MSB group which was likely due to hemoconcentration (*P* < 0.05–< 0.01). The A: G ratio was increased significantly in the MSB group due to the increased Hct. The toxic effects of MSB on broilers’ livers (Fig. 3D) were verified by elevated AST, ALT, and ALP levels (*P* < 0.05–<0.001).

**Fig. 4:**
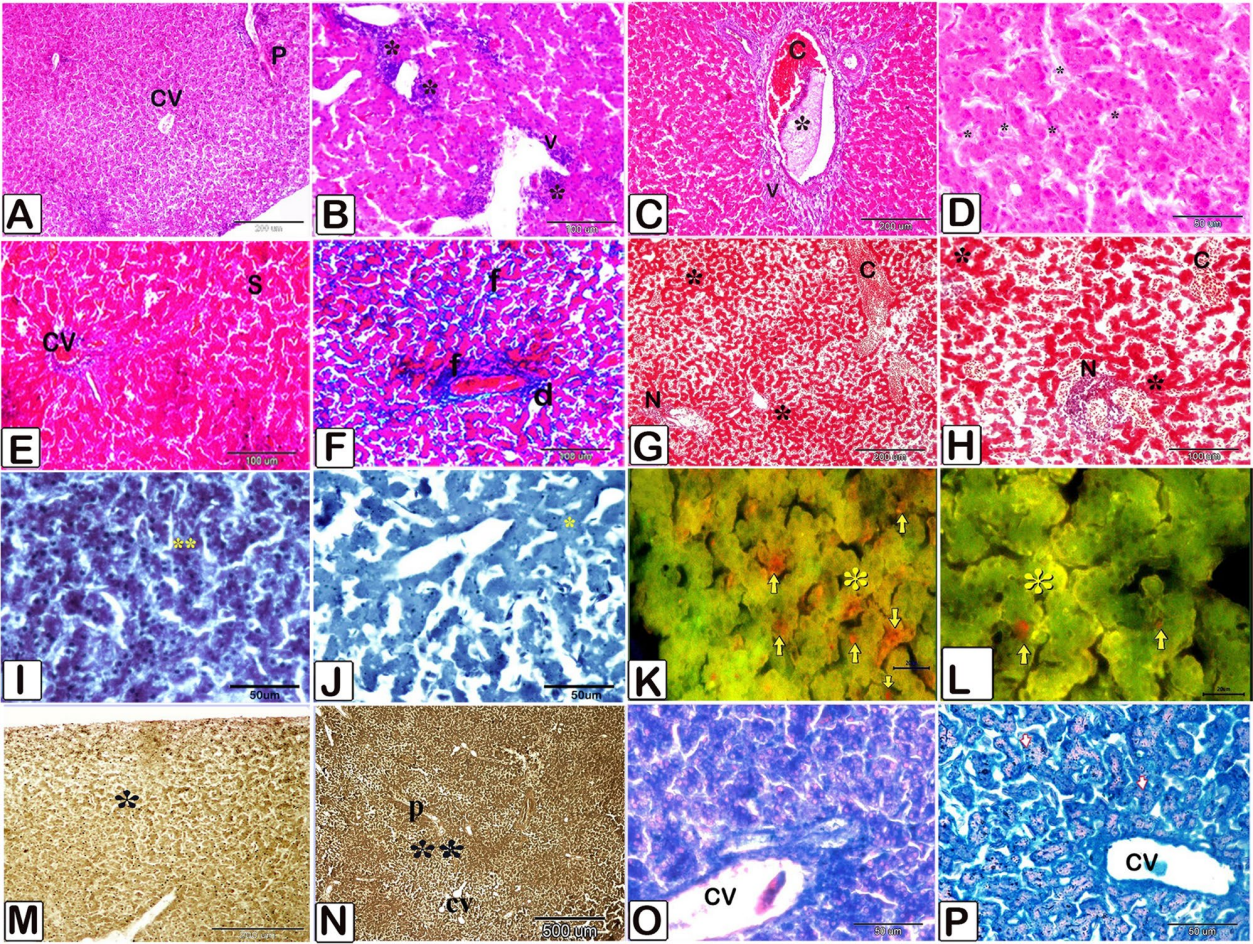
Liver histopathology including photomicrograph of Hematoxylin and Eosin (**A**-**D**), Crossmon’s trichrome (**E**, **F**), Ziehl-Neelsen (**G**, **H**), Mercury bromophenol blue (**I**, **J**), Acridine orange (**K**, **L**), Gomori calcium method (**M**, **N**), Prussian blue (**O**, **P**) stained paraffin sections of the liver from broilers exposed to MSB and free burning event. **A**: liver sample of the control group showed normal hepatocytes and no leukocytic infiltration. Note central vein (CV) and portal area (**P**). **B**, **C**, **D**: liver of the MSB group. **B**, **C**: lung of the MSB group showing vasculitis (v) and perivascular vascular lymphatic or leukocytic infiltration (*). **C**: embolic masses (asterisk), and vasculitis (V) of the blood vessels of the portal area, Venous congestion (**C**), **D**: embolic masses (asterisk) in the blood sinusoids. **E**: Control group showing central vein (CV) and liver sinusoids (**S**) without fibrosis. **F**: Liver of the MSB group showing interstitial and perivascular fibrosis (**f**), dilatation (**d**) of the blood sinusoids. **G**, **H**: hepatocytes of the MSB group exhibited a strong positive reaction for Long Ziehl Nielson stain for Lipofuscin pigment (*). Congestion of the central vein (**C**), Nodular lymphatic infiltration (**N**). **I**: liver sample of the control group. Blue coloration indicated positive protein inclusions. Hepatocytes had a high (**) affinity for mercury bromophenol blue that indicate cellular inclusions of protein nature that was uniformly distributed in the hepatocytes. **J**: liver sample from MSB group was stained by mercury bromophenol blue. Hepatocytes had a low affinity (*) for bromophenol blue that was indicated by a marked decrease in protein inclusions of the hepatocytes. Considerable loss and uneven distribution of protein inclusions are detected. K: hepatocytes exhibited yellowish reaction (*) and numerous kupffer cells (arrows) stained red. **L**: hepatocytes exhibited intense yellowish reaction (*) and few Kupffer cells (arrows) stained red. **M**: Gomori calcium method-stained paraffin sections of the liver for the detection of alkaline phosphatase activity. The liver control sample showed that hepatocytes had low affinity for alkaline phosphatase. **N**: In a liver sample from MSB group, alkaline phosphatase activities of hepatocytes varied according to metabolic zonation of the liver the hepatocytes in the periportal zone (**P**) had less alkaline phosphatase activities than hepatocytes in the Centrilobular zones (**). **O**: Pearls Prussian blue-stained the paraffin sections of the liver for demonstration of hemosiderin pigments. Liver sample of the control group showing hepatocytes in the Centro-lobular zone free from hemosiderin pigments. **P**: In a liver sample from MSB group, hepatocytes in the Centro-lobular zone exhibited hemosiderin pigments (arrows). Note central vein (CV)

**Fig. 5:**
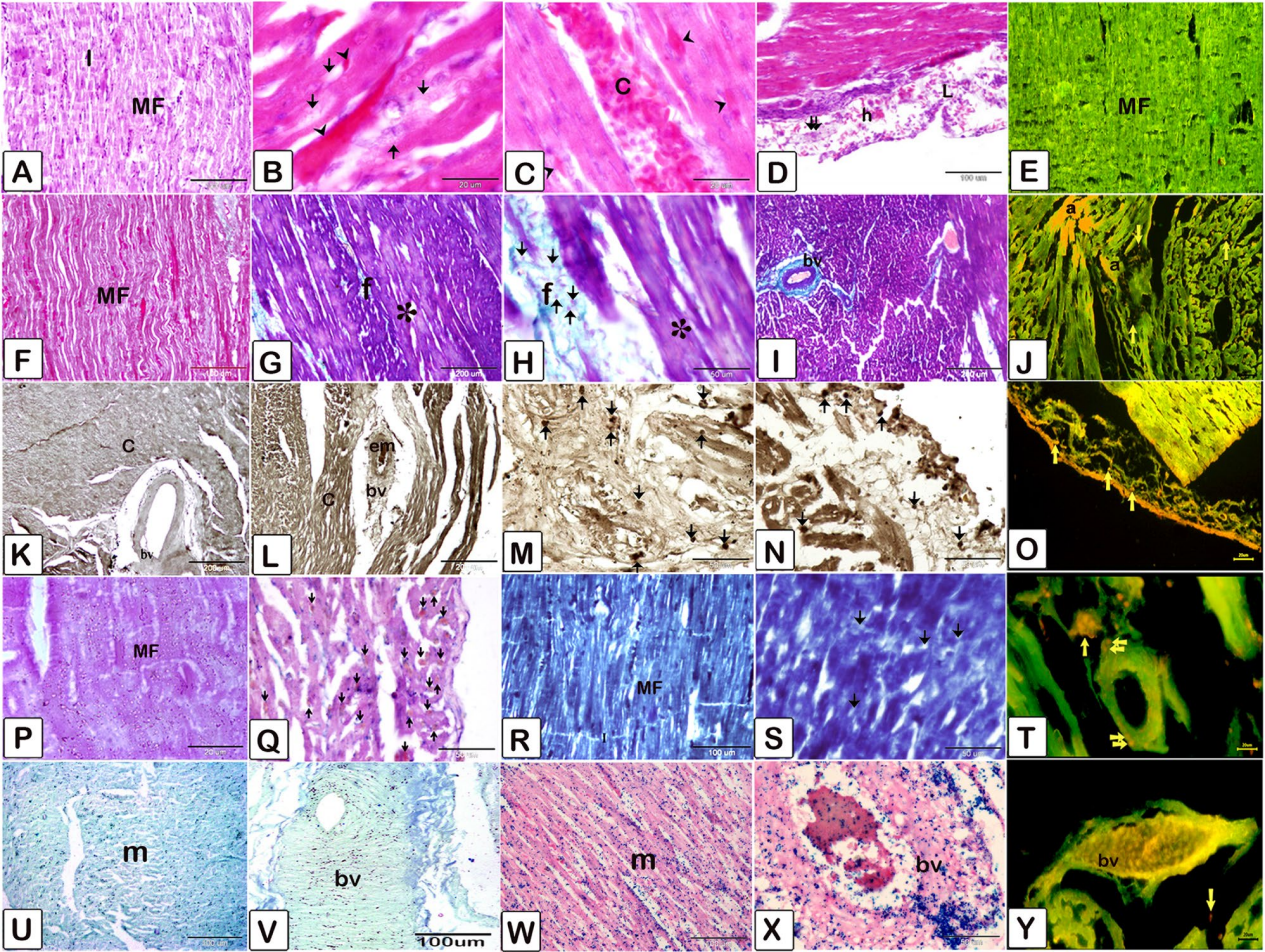
Heart Histopathology including photomicrograph of Hematoxylin and Eosin (**A**-**D**), Crossmon’s trichrome (**F**-**I**), Ziehl-Neelsen (**U**, **x**), Mercury bromophenol blue (**R**, **S**), Acridine orange (**E**, **J**, **O**, **T**, **Y**), Gomori calcium method (**K**-**N**), Prussian blue (**W**, **X**) stained paraffin sections of the heart from broilers exposed to MSB and control group. **A**: heart samples of the control group. Cardiomyocytes arranged in a branched pattern. Note myofilament (MF), intercalated disc (**I**). **B**, **C**, **D**: heart sample from MSB group. **B**: heart sample from MSB group, showed intense acidophilic cytoplasm (arrows), cytoplasmic vacuolations (arrowheads) of the necrotic cardiomyocytes **C**: Congested blood vessel (**C**), intense acidophilic cytoplasm of the necrotic cardiomyocytes (arrows) and destruction of myofilaments. **D**: Subendocardial lymphoid infiltration (**L**), and hemorrhage (**h**), degenerated Purkinje cell fiber (double arrows). **F**: Control samples. Note cardiomyocytes contained myofilaments (MF). **G**, **H**, **I**: heart sample from MSB group, showed degradation (asterisks) of the myofilaments was identified as Pale stained area exhibiting low affinity for acid fuchsin-acridine orange (*). Fibrotic changes in the interstitial tissue (**f**). Inflammatory cells (arrows). **I**: the embolic events (*) were detected in the blood vessels and capillaries (**c**) and fibrotic changes (**f**). Hypertrophy of the vascular wall (bv) and fibrotic changes (**f**) were observed. **K**, control samples of the heart. Note cardiomyocytes (**C**), blood vessel (bv). **L**, **M**, **N**: heart sample from MSB group. **L**: cardiomyocytes (**C**) exhibited high affinity for alkaline phosphatase. The embolic change (em) was detected in the blood vessel (bv). Note muscular tunic of the blood vessel exhibited high affinity for alkaline phosphatase. **M**: vascular wall underwent degeneration (**d**) of the endothelial cells and atrophied muscular tunic (**m**). Note inflammatory cells (arrows) penetrating the wall of the blood vessel (bv). **N**: cardiomyocytes (**C**) exhibited high affinity for alkaline phosphatase. Infiltration of the inflammatory cells (arrows) in the subendocardium and between cardiomyocytes. **R**: control samples, note cardiomyocytes contained myofilaments (MF), intercalated disk (**I**). **S**: heart sample from MSB group. Due to degenerative(**d**) processes myofilaments appear as dark homogenous areas. Areas of the cytoplasm of the cardiomyocytes exhibited complete destruction myofilaments (asterisks). **P**: control samples. Note cardiomyocytes contained myofilaments (MF without Lipofuscin pigments), and intercalated disk (**I**). **Q**: heart sample from MSB group. Lipofuscin pigments (arrows) were detected using Long Ziehl -Nielson for pigment detection. **U**, **V** represent the control group, and W, X represent the MSB group: hemosiderin pigments (arrows) were found in the cardiomyocytes of a cardiac sample from the MSB group. **E**, control samples of the heart showed cardiomyocytes (**C**) stained green with Acridine orange method. **J**, **O**, **T**, **Y**: Acridine orange method for detection of lysosomes from the heart sample from MSB group. **J**: interstitial inflammatory cells exhibited a yellow color indicating lysosomes persences. Note: lysosome-rich cardiomyocytes (**a**) stained yellow indicating apoptosis. **O**: endocarditis was identified by inflammatory cells rich in lysosomes (arrows). **T**: endomysial connective tissue was infiltrated by lysosome-rich inflammatory cells (arrows). Note inflammatory cells (double arrows) migrate through the wall of the blood vessels. **Y**: lysosome-rich inflammatory cells (arrows) in the congested blood vessel (bv)

**Fig. 6:**
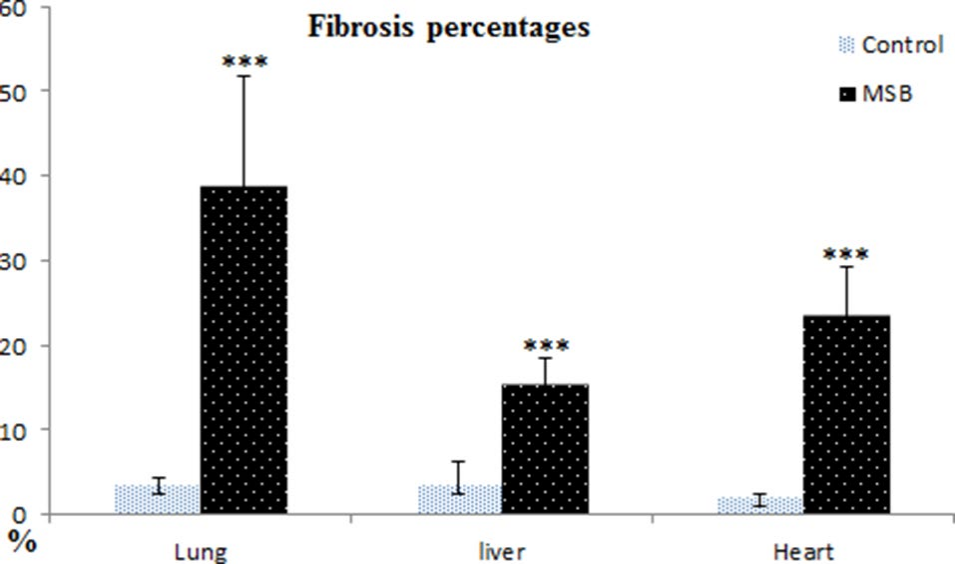
Collagen fiber percentage calculated from lung, liver and heart slides section stained by crossomon’s trichrome. Fibrosis percentages applied using Image free software (Fiji software Image J) (http://fiji.sc/Fiji). * Significant difference *P* < 0.05, ** highly significant difference *P* < 0.01, *** highly significant difference *P* < 0.001

The lungs of chickens (Fig. 4) exposed to MSB exhibited interstitial pneumonia with nodular and diffuse mononuclear inflammatory cell infiltration and hemorrhage. The lungs lost their inherent architecture and exhibited large necrotic areas, and fibrotic changes and elevated ALP levels. Histolpatholgical features of Liver tissue (Fig. 5), from the MSB group exhibited vasculitis and perivascular lymphatic or leukocytic infiltration, fibrotic changes and large populations of activated Kupffer cells as compared to the control groups. Histopathological features of heart tissues (Fig. 6), from the MSB groups exhibited carditis. Cardiomyocytes had evidence of degeneration including an intense acidophilic cytoplasm, cytoplasmic vacuolations, necrosis and lymphoid infiltration. Degradation of the myofilaments and fibrotic changes and were rich in ALP levels and lysosomes indicating apoptosis. Embolic events were identified in the blood vessels accompanied by hypertrophy of the vascular wall congestion hemorrhage. (Detailed result in supplementary file)

**Fig. 7:**
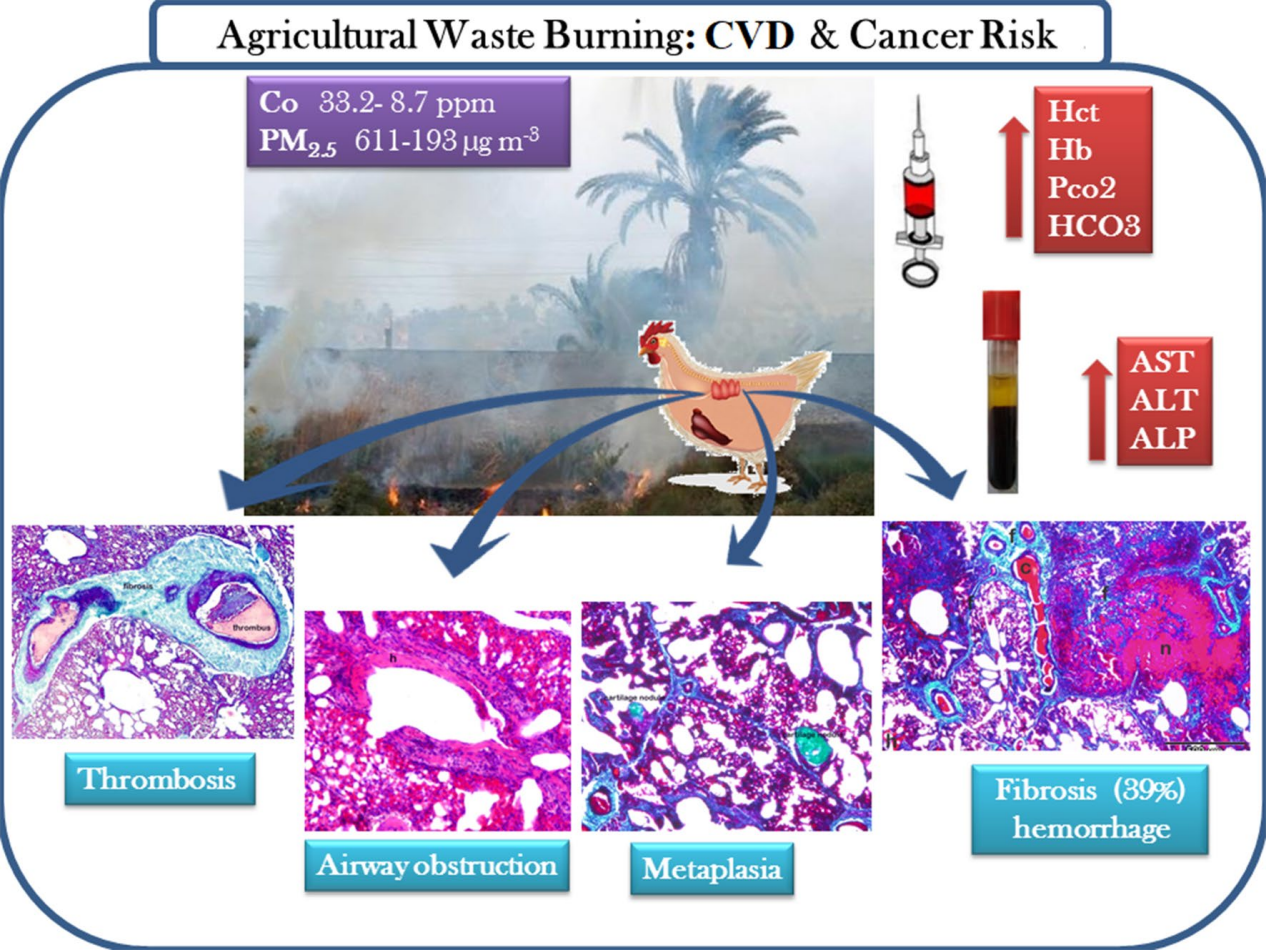
Illustration summary for seasonal agriculture burning effect on environment and health

Fibrous tissue percentages for lung hepatic and heart collagen fiber percentage were presented in Fig. 7. Fibrosis was estimated to be 38.8%, 23.5%, and 15.4% for the lung, heart, and liver, respectively. Highly significant differences were reported for fibrous tissue formation from lung (*P* < 0.01) which highlights the impairment of respiratory function.

## Discussion

CO concentration during MSB season was 15.4 ± 6.8 ppm. This is higher than the levels of 10 ppm and 6 ppm, stated by the Egyptian Environmental Affairs Agency (EEAA) and the WHO, respectively [17, 18], CO level in this study was remarkably higher compared to the level obtained from open rice straw burning (0.2–1 ppm) in Thailand [15]. Reports from a routine hourly analysis of 17 pollution stations around greater Cairo (3.3–8.5 mg/m3) emphasized the role of potential sources and weather in pollution [17]. However, there is a lack of data for the total emission of CO produced from the annual straw burning throughout Egypt. An increase in CO emission from bio burning could contribute to global warming [16]. Cases of poisoning have been reported from inhalation of even a small amount of CO in the open air [19]. This may be attributed to the greater affinity of CO (210 times) for Hb than oxygen resulting in cellular hypoxia, binding to myoglobin in the myocardium and impairment of the oxygen supply to mitochondria [20]. Tissue hypoxia results in platelets releasing nitric acid and the formation of the free radical, peroxynitrate [21]. Oxidative phosphorylation could be negatively affected, reducing the energy source for the heart and increasing the risk of arrhythmias and death from heart attacks [20].

Biomass open burning is one of the major sources of PM _2.5_ emissions which is accompanied by haze episodes in Assiut city and neighboring areas and characterized by high relative humidity and poor visibility for > 6 h leading to an increase in road accidents. In this study a concentration of PM_2.5_ was 301 ± 98 µg/m^–3^ which was 12 times higher than the level of 25 µg/m^–3^ recommended by the WHO [18]. PM_2.5_ was twice the level (134 µg/m^–3^) detected in Shanghai [22]. In Hungary and Romania, Particulate emissions from burning solid waste may account for as much as a few percent of the mass concentrations of PM_10_ in the atmosphere [23]. Dennis et al., 2002 displayed that 131, 203 acres of corn in Texas (USA) was burnt in 1996–1997, and the activities led 2502 short tons of PM2.5 (PM *<* 2.5 μm) per year. The highest average PM_2.5_ level (216.1 ± 11 µ/m^− 3^) was recorded in Cairo was dominated by open burning of trash and oil, and it exceeded the 24-h average US standard of 150 µg/m^–3^ at nearly all sites [24]. The PM_2.5_ concentration of greater Cairo was 85 and 70 µg m^–3^with a marked increase in the level due to rice straw burning [25]. Intensive MSB located west or northwest, in the upwind direction of Assiut, could enhance the transportation of emitted pollutants toward the city. The smoke could significantly contribute to increasing air pollution levels in the surrounding areas due to extra dust sources such as Assiut, is a sandwiched between two mountains. Pollutant emissions verified an upward trend from 2017 onwards, signifying an expansion in crop waste burning incidences [1].

BGA was used as a standard test for interpreting respiratory, circulatory, and metabolic disorders of the body [26]. Both CB and MSB broilers were apparently healthy. Therefore, BGA was used to assess the severity of respiratory compromise due to burning exposure as BGA has a minimal role in the diagnosis of CO poisoning [27]. CoHb should be measured by blood-gas analyzers with a CO-oximeter allowing for direct measurement. Unfortunately, this was not available in our study. The correlation between the appearance of symptoms and the level CoHb is doubtful. Extensive clinical reports have noted a lack of correlation between CoHb and the severity of CO poisoning suggesting that patients with a normal BGA, acidosis, or alkalosis could have equally severe neurologic symptoms at CoHb levels from 1 to 62% [28]. A similar result for BGA was reported in broilers exposed to stress in fast-growing strain experiments [29]. Higher levels of Hb and Hct were found in MSB broilers which reflected chronic stress conditions. They suggested that high Hb and Hct in broilers under extreme stress conditions could be a response to elevated erythropoiesis as a compensatory mechanism for O_2_ shortage in the tissue [30]. Elevated Hct was often associated with decreased bleeding and increased thrombus formation through the acceleration of platelet accumulation [31]. Short-term exposure to PM_2.5_ is accompanied by morbidity and mortality risk due to cardiovascular disease. Higher levels of PM_2.5_ were responsible for elevated blood coagulability and hypertension [28] and associated with higher blood pressure [35]. Liu et al. provided evidence that constituent of PM2.5 (EC, OC, NO_3_^–^, NH_4_^+^, and SO_4_^2–^) is responsible for increasing systemic blood coagulation, inflammation and hypertension induction [32].

Similarly, elevated PCO_2_ and HCO_3_ levels along with reduced PO_2_ levels were observed in fast-growing broiler experiments which may be related to hypoxemia and circulatory insufficiency resulting from a complex combination of circulatory failure and severe malfunction of gas exchange in the lungs [33]. ALT, AST, ALP, and TB were included in this study as biomarkers for tissue injury due to bio burning exposure, as these enzymes enter the bloodstream after cellular damage. ALT and ALP are produced primarily in the liver, so their increase could be considered the hallmark for detecting and classifying liver and bile duct damage, respectively [34, 35]. ALP hydrolyzes phosphate monoesters, which are required for a variety of cellular processes. However, ALT and AST are responsible for the transamination of alanine and aspartate which are found predominantly in the liver and extrahepatic organs (heart, lung, and muscle) [36]. A highly significant increase in ALT, ALP and AST were identified in this study which indicate profuse damage to the liver, bile duct, and other extrahepatic tissues, which was supported by the histopathological findings. Tissue damage due to xenobiotic contaminant exposure is a common event, like findings following increased in cyanide and carbon tetrachloride exposure suggesting hepatocyte damage accompanied by/with necrosis or alterations in cell membrane permeability, resulting in liver impairment function in broilers [37, 38].

MSB results in the release of CO and fine PM which in turn result in severe pneumonia, hepatitis, and carditis that may be identified by nodular and diffuse forms of mononuclear inflammatory cell infiltrations. CO directly destroys lung tissue prior to the formation of the COHb which impairs the capillaries permeability resulting in the leakage of macromolecules out of the blood vessels and hypoxia [39]. Metaplastic lesions occurs by the transformation of pulmonary fibrous tissue into cartilaginous nodules which may be considered a premalignant stage of cancer [40]. Fibrocartilaginous metaplasia may occur because of unbalanced regulation between growth factors, particularly TGF beta (fibroblast cell lineage) and Sox9 (chondrogenic lineage), and could be associated with the expression of types I and II collagen, S-100 protein, and chondroitin sulfate [41]. Epithelial-mesenchymal transition (EMT) as a response to PM2.5 metaplasia could occur due to excessive ROS, and certain components of PM_2.5_, could activate singling pathways that alter cytoskeletal origination [42]. In the current study, MSB exposed tissue exhibited a low affinity for elastic stains indicating the loss of elasticity required for respiration. Repair of lung elasticity following interstitial pulmonary damage requires further studies to explore the ability of pulmonary fibroblasts to secrete elastic fibers.

In this study fibrosis was estimated to be 38.8%, 23.5%, and 15.4% in the lung, heart, and liver, respectively. Extensive lung damage could be attributed to the direct deposition of PM_2.5_ in the lung tissue rather than deposition via systemic circulation [43]. Yoo et al., [44] found a 29.6% incidence of lung cancer in the fibrotic areas and a 44.4% incidence of fibrosis. In this contest, epithelial cells fail to regenerate which in turn activates growth factors leading to fibroblast accumulation, rapid epithelial proliferation, hyperplasia, and metaplasia. Epithelial activation acts as a nidus for lung cancer development [45].

The fibrotic changes found in this research in the heart and the liver were limited and likely insufficient to induce oncogenesis. Up to 80–90% of cases with liver fibrosis develop liver cancer [46], while the progression of fibrosis to cancer in the heart is uncommon [47]. Accordingly, exposure to PM_2.5_ led to pulmonary fibrosis which is associated with epithelial degeneration and metaplastic changes of interstitial and epithelial tissue suggesting that MSB exposure may act as a potent risk factor leading to lung cancer.

Myocardial and vascular degeneration are associated with the infiltration of inflammatory cells causing carditis. PM2.5 exposure is related to atherosclerosis, cardiovascular mortality, ischemic heart disease, and ventricular arrhythmias [48]. The effects of PM2.5 particles on cardiac muscle occurs through particles reaching the bloodstream and spreading directly to the heart and through the oxidative stress and inflammatory reactions [49]. PM2.5 exposure is responsible for inducing thrombogenic events which may be related to the loss of homeostasis in the pro-thrombotic/pro-coagulative state and CVD risk. In addition, fine particulate matter (PM2.5) raises the risk of respiratory and cardiovascular conditions, which cause over four million premature deaths annually worldwide [50].

**Fig. 8:**
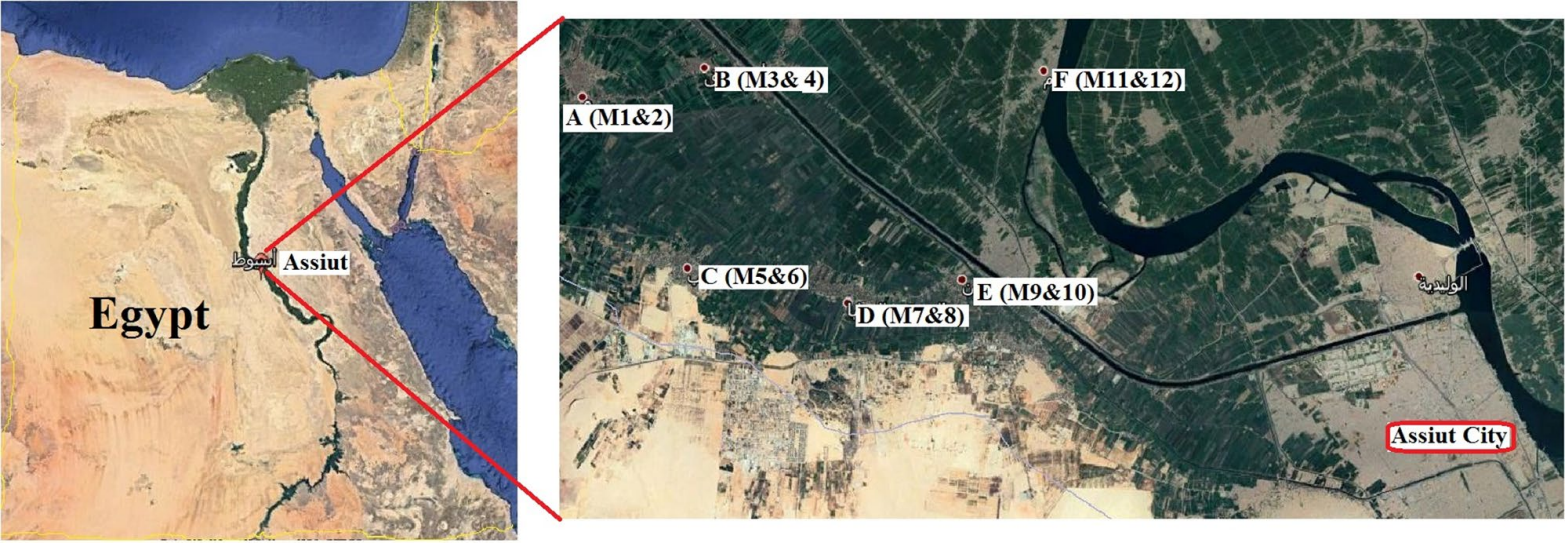
Sampling collection sites (Air and broilers) from six villages in Assiut, Egypt. **A**: Masraa village sample (M1 and M2). **B**: Awlad Rayek village samples (M3 and M4). **C**: Bani-ghaleb village samples (M5 and M6). **D**: Elbora and Alhedaya village samples (M7 and M8). **E**: Elwan village samples (M9 and M10). **F**: Sallam village samples (M11 and M13)

Increased ALP activity in the lung, liver, and heart following chronic MSB exposure was supported by serum analysis result. Although the exact mechanism for these health complications is still unclear, particle size, shape, number, and chemical composition are clearly important [51]. PM_2.5_ could pass through the respiratory system, reach the blood stream, and be deposited along the endothelial walls and within several organs thus exerting severe damage throughout the body. Illustration summery for environmental and health implications of the study was represented in Fig. 8.

Throughout 2003 and 2019, burning of agricultural waste resulted in 44,000 to 98,000 premature fatalities each year in India as result of the cultivation of residue-intensive crops, and a comparatively high downwind population density [52].

Agricultural waste management requires an integrated strategy that combines legislative support, community involvement, and technology innovation to mitigate the negative consequences of burning crop leftovers and encourage sustainable farming methods [53]. As an alternative of burning crop residue, composting, producing biofuel or using them as animal fodder could be explored.

## Conclusion

Sever environmental pollution as well as pneumonia, carditis and hepatitis were observed following MSB exposure. The lungs were the main organ affected with pulmonary fibrosis and metaplastic changes, airway obstruction and diminished respiratory performance. Embolic events in the vascular tissue of the lung, heart and liver indicted systemic embolism and cardiovascular disease risk. Exposure to agriculture waste burning is a potential hazard to livestock and human health. Our findings demand farmer education and immediate and active legislative measure against long-term crop residue management posing substantial economic, environmental and health risks that is frequently used in Egypt which continues because of financial limitations and restricted access to substitute technology in spite of its numerous detrimental effects. Further research could be compulsory comprising long-term monitoring of annual exposure and biomarker levels in different livestock and human to estimate the degree of risk for burning exposure.

## Materials and methods

The study protocol and procedures of the experiment were approved by the Animal Care and Use Committee Guide of the Faculty of Veterinary Medicine, Assiut University (Vet 129–2019). All procedures were performed in accordance with the relevant guidelines and regulations. The study was carried out in compliance with the ARRIVE guidelines (https://arriveguidelines.org) [54].

### Study area

The study was conducted during the seasonal maize stover (corn straw) burning after the peak harvesting season, which occurs during the autumn (late September to the middle of November) each year in Assiut (Governorate of South Egypt). Burning starts daily from 4 to 7 pm, in the villages and smoke can travel long distances based on wind direction and speed and can reach the urban areas of Assiut resulting in profuse clouds that affect vision, particularly on the roads, as well as a noxious smell. Private farms (3000–5000 bird capacity each) from six villages exposed to MSB within Assiut were involved in the study (Fig. 8).

### CO and PM2.5 measurements

A total of 36 air samples (three samples from each broiler farm) were collected for the determination of CO using oil-less compressor. Additionally, 12 air samples were collected from the same farms in free burning event which considered as control. Air samples were transported to the analytical chemistry laboratory of the Faculty of Science, Assiut University and CO levels were determined using an infrared detecting Multigas analyzer (ADC MGA-3000 series, USA). The flow rate (200 ml min–1) of air into the analyzer was controlled by a Dwyer mass flow controller (series GFC, USA).

TSI DustTrak II (8532) handheld Aerosol Monitors were used for PM evaluation for particle size > 0.1 μm. from studying poultry farms.

### Bird collection

A total of 60 apparently healthy broilers at the age of marketing (about 30 to 35 days old) were purchased from the exposed farms, at the end of October 2019. The controls were collected in December, well after the burning season. MSB and control broilers (CB) were transported to the Animal Study Unit at the Faculty of Veterinary Medicine, Assiut University, Egypt.

### Blood-gas analysis (BGA) and serum sample

Birds were sedated with sodium pentobarbital (30 mg/mL), Blood samples were collected by cardiac stab into heparinized monovette syringes. Each bird was placed on its right side, and the needle was judged to have pierced the left ventricle, and was sent immediately to the laboratory for pH, pCO2, pO2, hematocrit (Hct), and Hb using a Radiometer ABL 800 Basic device. Serum samples were collected for Total protein (TP), total bilirubin (TB), ALT, AST, and ALP measurement spectrophotometrically using commercial diagnostic kits obtained from Vitro Scient Co. (Egypt). Birds were euthanized immediately following blood collection through cervical dislocation. A mid-line incision along the thoracic inlet was made, and liver, lungs, and heart were collected for histopathological examination.

### Histopathology

On the department of cell and tissues, Faculty of Vet Medicine, samples were processed for paraffin embedding methods according to the technique described by Abd-El-Hafeez et al., [55]and were stained by the Hematoxylin and Eosin (H&E), histochemical stain: Crossomon’s trichrome, bromophenol blue stain, Wiegert’s stain, Vân Gieson, Prussian blue stain, Long Zheil Nielsen stain Histochemical analysis of Alkaline phosphatase [56] examined by light microscope. Acridine Orange was performed according to Hoff et al., Mahmoud et al., [57], Abd-El-Hafeez et al. [58]and examined by fluorescent microscope.

### Estimation of fibrous tissue percentage (F%)

Lung, hepatic and heart collagen fiber percentage were measured in slide section stained by crossomon’s trichrome using Image free software (Fiji software Image J) (http://fiji.sc/Fiji) according to [25, 59].

### Statistical analysis

Data were analyzed using one way of variance (ANOVA) using the SPSS 16 program for windows, version 5.0 (San Diago CA. USA). Data were expressed as mean ± standard error (SE). The significant difference between MSE broilers and control was accepted at *p* < 0.05, 0.01 or 0.001.

## Electronic supplementary material

Below is the link to the electronic supplementary material.

Supplementary Material 1

## Data Availability

The datasets used and/or analyzed during the current study are available from the corresponding author on reasonable request.
