# Supplementary material for: Maize stover burning exposure accountable for remarkable environmental and health risk in broiler chickens
Source: BMC Vet Res. 2025 Mar 25;21:199. doi: 10.1186/s12917-025-04476-7 (PMC11934768; doi:10.1186/s12917-025-04476-7)
Supplement: Supplementary file 1 — Supplementary Material 1 [file 12917_2025_4476_MOESM1_ESM.docx]

Supplementary file

**Agricultural waste burning exposure accountable for remarkable environmental and health risk in poultry**

**Manal A. M. Mahmoud^*1^_,_ Abd El-Aziz A. Said^2^, Hanan H. Abd- Elhafeez^3^, Soha A. Soliman^4^, Usama T. Mahmoud^5^**

^1^Department of Animal Hygiene, Faculty of Veterinary Medicine, Assiut University, Assiut 71526, Egypt**.**

^2^ Chemistry Department, Faculty of Science, Assiut University, Assiut 71526, Egypt

3 Department of Cell and Tissues, Faculty of Veterinary Medicine, Assiut University, Assiut 71526, Egypt

4 Department of Histology, Faculty of Veterinary Medicine, South Valley University, Qena 83523, Egypt

^5^Department of Animal, Poultry and aquatic life Behaviour and Management, Faculty of Veterinary Medicine, Assiut University, Assiut 71526, Egypt

*Corresponding: manalmahmoud@aun.edu.eg

1. **Materials and methods**

The study protocol and procedures were performed in accordance with the Animal Care and Use Committee Guide of the Faculty of Veterinary Medicine, Assiut University, Egypt.

- 1. **Study area**

The study was conducted during the seasonal maize Stover (leftover corn straw) burning after the peak harvesting season, which occurs during the autumn (late September to the middle of November) each year in Assiut (Upper Egypt). Burning time in the study area occurs daily, from 4–7 pm, in the villages and neighboring areas. Smoke can travel long distances based on wind direction and speed and can reach the urban areas of Assiut resulting in profuse clouds that affect vision, particularly on the roads, as well as a noxious smell with respiratory effects. Two private farms (3000–5000 bird capacity each) from six villages within Assiut with a high rate of maize stove burning were involved in the study (Figure 1).

- 1. **Carbon monoxide measurement**

A total of 36 air samples (three samples from each broiler farm) were collected for determination of ambient CO concentration using an oil-less compressor at ambient temperatures during October 2018 (broilers were 5–35 days old at that time) (Figure 1). Three measurements of each sample were taken. Samples were obtained between 6–10 pm corresponding to the peak of smoke in the area. Additionally, 12 air samples were collected from the same farms during the subsequent broiler cycle which occurred without any burning and were considered to be control samples. Air samples were transported to the analytical chemistry laboratory of the Faculty of Science, Assiut University and CO levels were determined using an infrared detecting Multigas analyzer (ADC MGA-3000 series, USA). The flow rate (200 ml min^–1^) of air into the analyzer was controlled by a Dwyer mass flow controller (series GFC, USA).

- 1. **Particulate matter evaluation**

TSI DustTrak II (8532) handheld Aerosol Monitors were used for small PM evaluation for particle size >0.1 µm. PM levels were recorded for three time periods during the MSB season (beginning, middle, and end) at the 12 poultry farms studied. A total of 84 PM values were recorded including 12 taken one month after the burning season. The apparatus was calibrated every 2 weeks, and each recorded value was replicated 3 times to ensure accuracy.

**2.4 Bird collection**

A total of 60 apparently healthy broilers, 5 from each of the 12 farms, about 30 to 35 days old were collected at the end of October 2018. The controls were collected at the beginning of December, well after the burning season. Two control broilers from each farm, 24 birds altogether, were collected and subjected to the same procedures as the maize stove burning (MSB) exposed groups and analyzed together. MSB and control broilers (CB) were transported to the Animal Study Unit at the Faculty of Veterinary Medicine, Assiut University, Egypt where they were euthanized and samples were collected.

- 1. **Blood-gas analysis (BGA)**

Blood samples were collected by cardiac stab into heparinized monovette syringes. Each bird was placed on its right side, and the needle was inserted between the third and fourth true ribs. The needle was judged to have pierced the left ventricle, based on the bright red color of the blood, and was sent immediately to the laboratory for analysis. Samples were analyzed for pH, pCO_2_, pO_2_, hematocrit (Hct), and Hb using a Radiometer ABL 800 Basic device. The primary blood values for pH, PCO_2_, and PO_2_ were generated by the blood-gas analyzer operating at a sample chamber temperature of 37º C and were then recalculated by the ABL800 for a temperature of 41º C to match the normal body temperature of domestic fowl (Fedde, 1986).

**2.6. Serum sample collection for bilirubin and enzymes**

Birds were sedated with sodium pentobarbital (30 mg/mL), and 10 mL of blood was collected via cardiac puncture into a serum separator tube without anticoagulant (FL Medical, Italy). Birds were euthanized immediately following blood collection through cervical dislocation. A mid-line incision along the thoracic inlet was made, and liver, lungs, and heart were collected for histopathological examination. Serum total protein (TP), albumin (A), globulin (G), total bilirubin (TB), alanine aminotransferase (ALT), aspartate aminotransferase (AST), and alkaline phosphatase (ALP) were measured spectrophotometrically using commercial diagnostic kits obtained from Vitro Scient Co. (Egypt) according to the manufacturer’s instructions. The serum samples from each farm were pooled and analyzed together.

- 1. **Histopathology:**

Each organ was divided into two groups. A specimen (one cm^3^) of the first group was fixed in Bouin’s solution and processed for paraffin embedding blocks and sections. Another specimen was kept at (-20^o^C) and subjected to alkaline phosphatase enzyme detection.

- - 1. **Light microscopic examination:**
       1. **Sample processing for paraffin embedding, general and histochemical staining techniques:**

Samples were processed according to the following technique described by [Abd-Elhafeez and Soliman [15](#_ENREF_15)] Sections were prepared by using a Richert Leica RM 2125 Microtome, Germany and were stained by the Hematoxylin and Eosin (H&E), histochemical stain: trichrome according to Crossomon’s for collagenous fibers and to differentiate between constituents of the tissues, bromophenol blue stain for demonstration of total protein, Wiegert’s stain use to demonstrate loss of elastic fiber in counter by Van Gieson stain for collagen, the Prussian blue stain for iron demonstrates hemosiderin, lipofuscin pigments which were stained with Long Zheil Nielsen stain [[16](#_ENREF_16)].

- - - 1. **Enzyme histochemical stain using Gomori calcium method for alkaline phosphatase alkaline activity**

Histochemical analysis of Alkaline phosphatase was performed on frozen sections (10 μm) which obtained in Leica cryostat CM 1900-6-1 (Richert, Germany) and
stained according to the description by [[16](#_ENREF_16)]. Leitz Dialux 20 Microscope provided with a Canon digital camera (Canon Powershot A95) was used to examine the stained sections.

- - 1. **Fluorescent microscopic examination by using Acridine Orange (Fluorescent stain)**

Acridine Orange is employed for identification of the secretory vesicles, lysosomes and apoptosis. The procedure was performed according to that [Hoff, Newman [17](#_ENREF_17)] and [Mahmoud, Zaki [18](#_ENREF_18)]. The stained sections were analyzed using a microscope model Letiz DM 2500 with external fluorescent unit Leica EL 6000 at Anatomy, Embryology and Histology Department, Faculty of Vet Medicine, Assiut, Egypt.

**3. Results of the manuscript**

CO levels during MSB season ranged from 8.7–33.2 ppm (mean, 15.41 ± 6.8). There was no significant difference between the three CO levels taken at each farm during MSB season. However, control samples from the beginning of December were 1.13 ± 1.002 ppm (Figure 2A). Fine PM_2.5_ was significantly higher during the MSB season than the control season. The highest PM_2.5_ level was 611µg/m^–3^ and the lowest was 193 µg/m^–3^ (mean, 301 ± 98 µg/m^–3^) during MSB season. During the control period the PM_2.5_ concentrations were as low as 64 ± 19 (Figure 2B). The effects of maize burn exposure on pH and blood gases are noted in Figure 3A. The broilers’ pH was not affected by MSB. However, pCO_2_ and HCO_3_ levels were increased, and pO_2_ was decreased compared to controls. The Hb and Hct were significantly increased in MSB broilers compared to control.

The broilers biochemical parameters are shown in Figures 3B, C, D. TB (direct and indirect) and TP (Figures 3B, C) did not differ significantly between the MSB and control groups. However, there was a significant decrease in G and increase in A in the MSB group which was likely due to hemoconcentration (*P* <0.05–< 0.01). The A:G ratio was increased significantly in the MSB group due to the increased Hct. The toxic effects of MSB on broilers’ livers (Figure 3D) were verified by elevated AST, ALT, and ALP levels (*P* <0.05–<0.001).

The lungs of chickens exposed to MSB exhibited interstitial pneumonia with nodular and diffuse mononuclear inflammatory cell infiltration (Fig. 4 B–D, H). Lung tissue exhibited hemorrhage (Fig. 4B–D) and congested capillaries (Fig. 4 B–D, F) while muscle fibers underwent hyalinization (Fig. 4D). The lungs lost their inherent architecture and exhibited large necrotic areas and fibrotic changes (Fig. 4D, F). Lipofuscin pigment was detected in interstitial cells using Ziehl-Neelsen staining (Fig. 4G, H). Pneumocytes had a low affinity for bromophenol blue due to a marked decrease in protein inclusions, and there was considerable loss and uneven distribution of protein inclusions (Fig. 4I, J). Lung tissue exposed to MSB exhibited elevated ALP levels (Fig. 4K, L), and necrotic areas exhibited hemosiderin and lipofuscin pigmentation (Fig. 4M, N). Decreased lung macrophage activity was observed (Fig. 4O, P). The lungs of the control group (Fig. 4 Q, R) had an abundant amount of elastic tissue in the interstitium, while the lungs of the MSB group had scant fibro-elastic tissue in the interstitium (Figure 4S, T).

Liver samples from the MSB group exhibited vasculitis (Fig. 5B, C) and perivascular lymphatic or leukocytic infiltration was common (Fig. 5B, C). Fibrotic changes were detected by Crossman’s trichrome (Fig. 5E, F). Hepatocytes exhibited a strong positive reaction to Long Ziehl Nielson stain for lipofuscin pigment. Nodular and diffuse mononuclear inflammatory cell infiltrations were detected in the portal area (Fig. 5G, H). Hepatocytes from the MSB group exhibited a low affinity for mercury bromophenol blue (Fig. 5J) compared to hepatocytes from the control group (Fig. 5I). Hepatocytes from the MSB group exhibited elevated ALP activity (Fig. 5L) when compared the control group (Fig. 5K) using the Gomori calcium method. Hepatocytes from the MSB group had hemosiderin pigments (Fig. 5N), using Pearls Prussian blue, as opposed to the control group which did not (Fig. 5M). Liver samples from the control group had large populations of activated Kupffer cells (Fig. 5P) as compared to the MSB groups (Fig. 5O).

Heart samples from the MSB groups exhibited carditis. Cardiomyocytes had evidence of degeneration including an intense acidophilic cytoplasm, cytoplasmic vacuolations, necrosis (Fig. 6A-C), lymphoid infiltration (Fig. 6D, M, N), degradation of the myofilaments (Fig. 6B-C, G, R, S), and fibrotic changes (Fig. 6G, H). Purkinje cell fibers underwent degeneration (Fig. 6D). Embolic events were identified in the blood vessels (Fig. 6J, L). Hypertrophy of the vascular wall (Fig. 6I) was noted, and the endothelial cells and atrophied muscular tunic of the vascular wall underwent degeneration (Fig. 6M). Vascular congestion (Fig. 6C), hemorrhage (Fig. 6D), and cardiomyocytes exhibited elevated ALP levels (Fig. 6K, L, N). The muscular tunic of the blood vessels also exhibited elevated ALP levels (Fig. 6L). Cardiomyocytes had lipofuscin pigments that were detected using Long Ziehl Nielson stain (Fig. 6P, Q) as well as hemosiderin pigments (Fig. 6 U-X). Cardiomyocytes were rich in lysosomes indicating apoptosis (Fig. 6J). Lysosome rich inflammatory cells were identified in the blood vessels and migrated to the perivascular tissue, endometrial tissue, and endocardium (Fig. 6J, O, T, Y). The average percentage of collagen fiber in the lung, liver, and heart (Figure 7) of the control and MSB groups was 38.8 ± 12.9%, 15.4 ± 3.1%, and 23.5 ± 5.7 %, respectively.

Fibrous tissue percentages for lung hepatic and, heart collagen fiber percentage were presented in figure 7. Fibrosis was estimated to be 38.8%, 23.5%, and 15.4% for the lung, heart, and liver, respectively. Highly significant differences were reported for fibrous tissue formation from lung (P< 0.01) which highlights the impairment of respiratory function.

**Additional Results and figures**

Lung of control group showing(Fig.1 A and B) showing the normal structure of parabronchus with atria continued with air capillaries without signs of inflammation. The lung of groups of burning maize exhibited interstitial pneumonia that had nodular and diffuse mononuclear inflammatory cell infiltrates (Fig. 1C, D, E-K). The pneumocytes of the air capillaries undergo hypertrophy and acquired cuboidal profile (Fig. 1D). SMF hyalinization occurred in (Fig. 1 G-J). The SMF of the atrial wall hypertrophied causing obstruction   of the respiratory airways (Fig. 1 G-J). The parabronchial epithelium undergoes hypertrophy (Fig. 1K). The lung also showed hemorrhage (Fig. 1D-K) and congestion of the blood vessels (Fig. 1D, E, F, I, Fig. 2C, D, H, J, Fig. 4E). The parabronchus loss their typical architecture that had no atria (Fig. 2 C, f, G, H).Necrotic masses were detected and pulmonary fibrosis was identified by Crosmon’s trichrome and the lung exhibited distinctive lobulation (Fig. 2C, f-K). The necrotic debris and the fibrous tissue replaced the collapsed air capillaries (Fig. 2K).  The atria were enlarged due to the formation of fibrotic tissue (Fig. 2D, E). The hyaline  cartilaginous nodule was formed in the parabronchial wall (Fig. 3A-C). Degradation of the cartilage of extrapulmonary bronchus occurred (Fig. 3D,F ) and inhomogeneous staining of the cartilage matrix (Fig. 3E).  The elastic tissue in the lung of the control group (fig. 3 G) are abundant in the interstitial tissue while the lung of the burning maize group had scant fibro-elastic tissue in the interstitial.  Using Ziehl-Neelsen, the lung of the control group (A, B) had no affinity for the stain in comparison to pneumoniatic lung of the groups of burning maize had lipofuscin pigment-positive inflammatory cell infiltrates that exhibited either nodular or diffuse form (Fig. 4D, E). lipofuscin pigment-positive reaction was also observed in other interstitial cells (Fig. 4 D, E), and the exfoliating cells in the parabronchus (Fig. 4C). Protein inclusions were detected using mercury bromophenol blue. In the control group, uniformly distributed staining of lung tissue including the pneumocytes indicated positive protein inclusions (Fig. 5A-C). Lung sample of groups of burning maize showing the pneumocytes had a low affinity for bromophenol blue that was indicated by a marked decrease in protein inclusions and the uneven distribution of protein inclusions in the lung tissue (Fig. 5D-F). Lung tissue including the pneumocytes and the macrophages exhibited high affinity for alkaline phosphatase activity in lung sample from groups of burning maize (Fig. 6D-F) compared with lung s of control group (Fig. 6A-C) using Gomori calcium method. Lung pneumocytes and the macrophages and the necrotic areas had hemosiderin pigments in lung sample from groups of burning maize (Fig. 7 C-E) compared with the lung of control group (Fig. 7A, B) using Pearls Prussian blue. acridine orange was used for detection of lysosomes-rich cells particularly lung macrophages.   lung sample of the control group had numerous activated lung macrophages (Fig. 8A, B) while low  alveolar macrophages activities were detected in the  lung sample of the groups of burning maize (Fig. 8C, D).

Liver samples of burning maize-exposed groups had vasculitis (Fig. 9B, C, F) in comparison to normal hepatocytes and no leukocytic infiltration of the control group (Fig. 9 A). Perivascular lymphatic or leukocytic infiltration was common (Fig. 9B, C) as well as nodular lymphatic infiltration was detected (Fig. 9E, H). congestion of the central vein and the blood sinusoids, dilation and obstruction of the blood sinusoids occurred (Fig. 9D, G, Fig. 11A, D, E). Embolic masses were observed in of the blood vessels of the portal area (Fig. 9F) and blood sinusoids (Fig. 9I, 10F). Necrotic cells were identified as acidophilic bodies (Fig. 9E, H). Liver samples of burning maize-exposed groups undergo fibrotic changes which were detected by Crossmon’s trichrome (Fig. 10B, C, G-I). Hepatocytes exhibited a strong positive reaction for Long zheil Nielson stain for Lipofuscin pigment. Nodular lymphatic infiltration, lymphocytic infiltrations were detected in the portal area (Fig. 11 A-F). Hepatocytes had a low affinity for mercury bromophenol blue in the liver sample from groups of burning maize (Fig. 12D, E, F) compared with Hepatocytes of control group (Fig. 12A, B,C). Hepatocytes exhibited high affinity for alkaline phosphatase activity in liver sample from groups of burning maize (Fig. 13E, F, G, H) compared with hepatocytes of the control group (Fig. 13A, B, C, D) using Gomori calcium method. Hepatocytes had hemosiderin pigments in liver sample from groups of burning maize (Fig. 14 C, D) compared with Hepatocytes of the control group (Fig. 14A, B) using Pearls Prussian blue stain. lysosomes activities of kupffer cells were estimated using Acridine orange. Liver sample of the control group had large populations of activated kupffer cells (Fig. 15 A, B) compared with a liver sample of the groups of burning maize (Fig. 15 C, D).

Heart samples of burning maize-exposed groups showed carditis. Cardiomyocytes had signs of degeneration including intense acidophilic cytoplasm, Cytoplasmic vacuolations, necrosis (Fig. 16 A, B, D-F, G, Fig. 17 A, B, C, H, Fig. 18 G-L), degradation of the myofilaments (Fig.18 H, L), lymphoid infiltration (Fig.16H, I, Fig. 17 H, Fig.20 C, D, F, I, K), fibrotic changes (Fig. 17 B, D, G-I). Purkinje cell fiber underwent degeneration (Fig. 16 C, H, I). Embolic events were identified in the blood vessels (Fig.17 D-F, Fig.20 E, H). Hypertrophy of the vascular wall (Fig. 17 I). The vascular wall underwent degeneration of the endothelial cells and atrophied muscular tunic. The inflammatory cells penetrating the wall of the blood vessel (Fig. 20 K). Vascular congestion was common (Fig. 16 G, Fig. 18 K). using Long zheil Nielson, Lipofuscin pigments appeared as granules (Fig. IIA, B) or distributed in the whole cardiomyocytes (Fig. 18 D), and in the blood vessels (Fig. 18 C). hemosiderin pigments were detected in the cardiomyocytes (Fig. 18 E, F). using Acridine orange, Cardiomyocytes were rich in lysosomes indicating apoptosis (Fig. 19 A,-D, F, G). lysosome-rich inflammatory cells were identified in the blood vessels and migrate to the perivascular tissue, endomysial tissue, endocardium (Fig. 19 C-I). Cardiomyocytes exhibited high affinity for alkaline phosphatase (Fig. 20 A-G, H). Muscular tunic of the blood vessel exhibited high affinity for alkaline phosphatase (Fig.20 E, H-J).

Figures and legends:


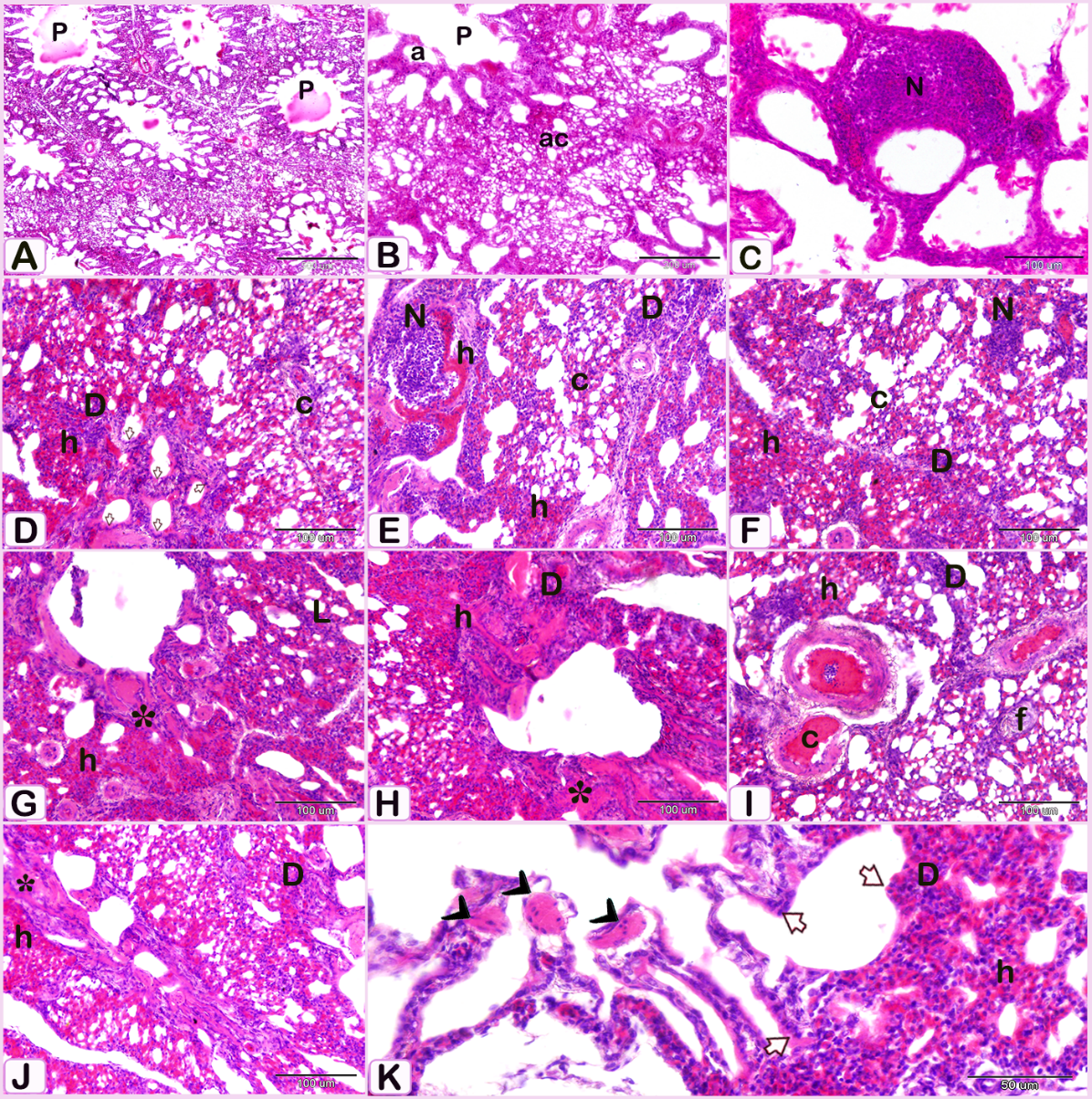


Fig 1:  paraffin sections of Lung were stained by Hematoxylin and Eosin (HE).A, B: the control group and C-K from groups of burning maize showing in

A, B:  lung of the control group. The atria (a) opened into the parabronchus (P). The air capillaries (ac) continued with the atrium. The structure is nornmal without hemorrhahge or inflammatorty cellular infiltration.

C, E, F, G:  nodular (N) and diffuse (D) mononuclear inflammatory cell infiltrates, Hemorrhage (h), congested blood capillaries (C).  D: Lung exhibited interstitial pneumonia. Note lymphocytic infiltrations (D), Hemorrhage (h), hypertrophy of the pneumocytes of the air capillaries and acquired cuboidal profile (arrows), congested blood ; (C).    G-J: hyalinized muscle fibers (asterisk in figure J), and numerous inflammatory cells.Hypertrophy of the SMF causing obstruction   of the respiratory airways( arrowheads in figure K). Hemorrhage (h), diffuse (D) mononuclear inflammatory cell infiltrates. pulmonary fibrosis (F). K: mononuclear (D) inflammatory cell infiltrations, hypertrophy of the parabronchial epithelium (arrows). Hemorrhage (h).


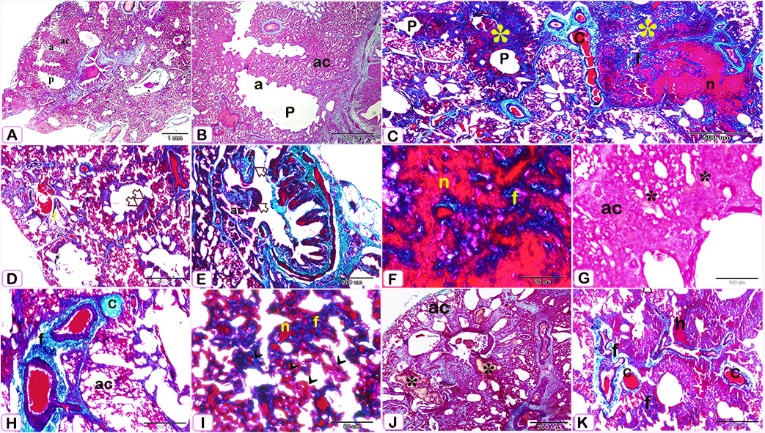


Fig. 2: paraffin sections of Lung were stained by Crosmon’s trichrome for detection of fibrosis. A, B: Lung of the control group.C-K Lung samples from burning maize group.

A, B: Lung of the control group showing the parabronchus (P). The atria (a) opened into the parabronchus. The air capillaries (ac) continued with the atrium.Normal structure of lung without fibrosis.

C, f, G: parabronchus (P) loss the architecture that had no atria. Pulmonary fibrosis (asterisks). Vascular congestion (C). Necrotic mass (n) surrounded by fibrous tissue (f). Collapsed air capillaries (ac). D, E: enlargement of the atria due to fibrotic changes (arrows). Fibrous tissue (f) replaced the air capillaries. Lung undergoes fibrosis that acquired distinctive lobulation. Congestion (c). H: pulmonary fibrosis (f). Formation of nodular masses of hyaline cartilage (c). Collapsed air c**a**pillaries (ac). I, J: necrotic masses and Fibrous tissue (f), Parabronchus (P) hemosiderin pigment (asterisks),hemorrhage(arrowheads). Congestion (c). k: Fibrous tissue (f) replaced the collapsed air capillaries (ac). Hemorrhage (h). congestion of the pulmonary vessels (c).

Dxz
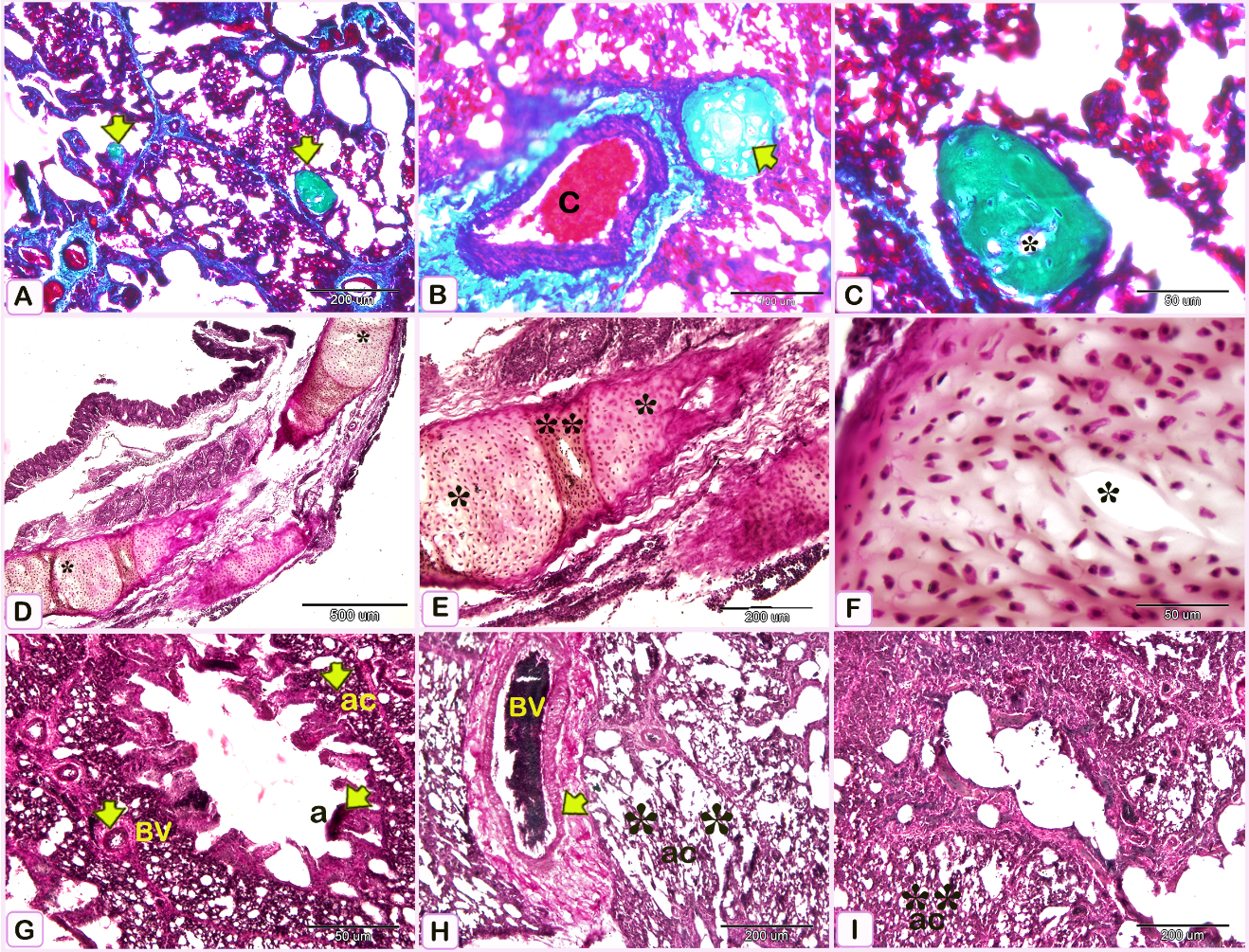


Fig 3: Formation of intrapulmonary cartilage nodules and degradation of extrapulmonary cartilage and Loss of elastic tissue of the lung.

Lung samples from burning maize group (A-f, H and I), figure (G) the lung sample from the control group. A_C: paraffin section stained by Crossmon trichrome.D_I: paraffin section stained by Weigert stain.

A-C: Formation of the Hyaline cartilaginous nodule (arrowheads). Note degradation of the cartilage matrix (asterisk). Note congested blood vessels (C). D, F: degradation of the cartilage of extrapulmonary bronchus (asterisks).E: inhomogeneous staining of cartilage matrix showing dark (**) and pale staining (*).G: lung of control group showing the abundant elastic fibers (ac,yellow arrows) around the air capillaries and in ateria (a, yellow arrow) and in wall of a blood vessel (BV, yellow arrow). H and I: Lung samples of the burning maize group showing reduce elastic fibers in the interstitial tissue and around air capillaries(**) and in-wall of a blood vessel (BV,yellow arrow).


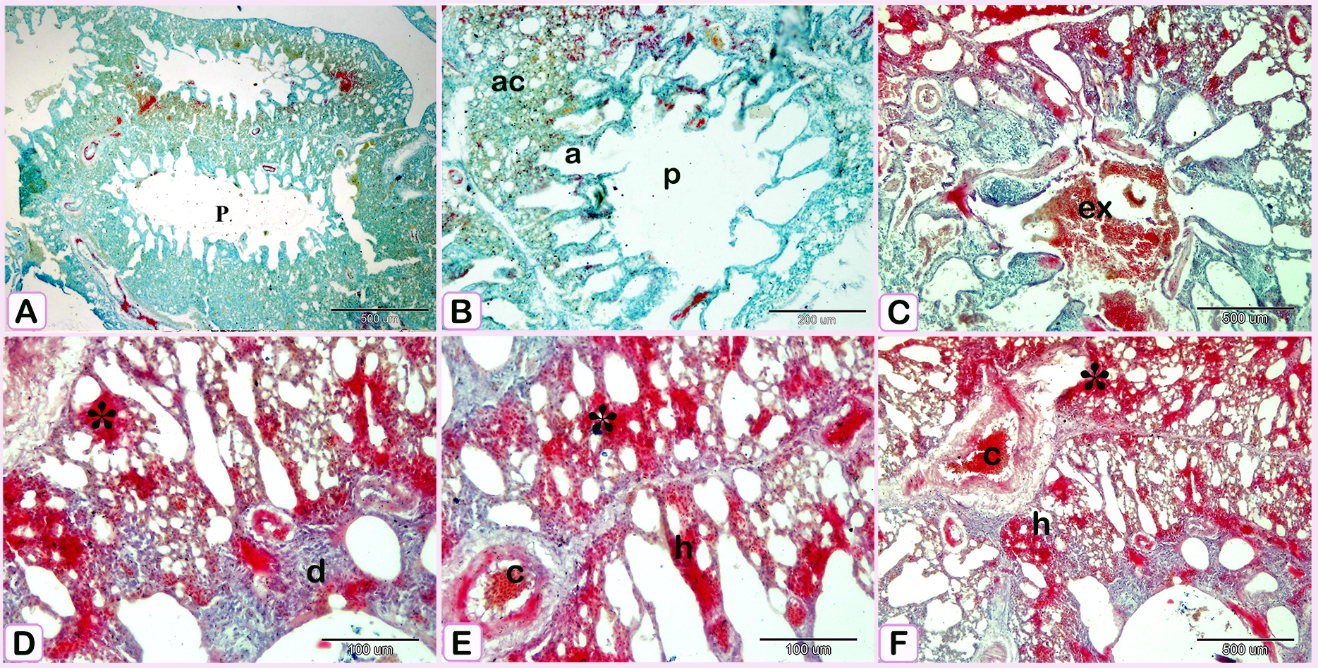


Fig 4: paraffin sections of Lung were stained by Long Zheil Nielsen stain for detection of lipofuscin pigments. A, B: Lung of the control group.C-F: Lung samples from burning maize group.

A, B: Lung of the control group showing no affinity for the stains. The atria (a) opened into the parabronchus(P). The air capillaries (ac) continued with the atrium. C: lipofuscin pigment positive exfoliating (ex) cells in the lumen of the parabronchus. D-F: lipofuscin pigment positive interstitial cells(*), Hemorrhage (h), vascular congestion (C), diffuse (d) inflammatory cell infiltrates.


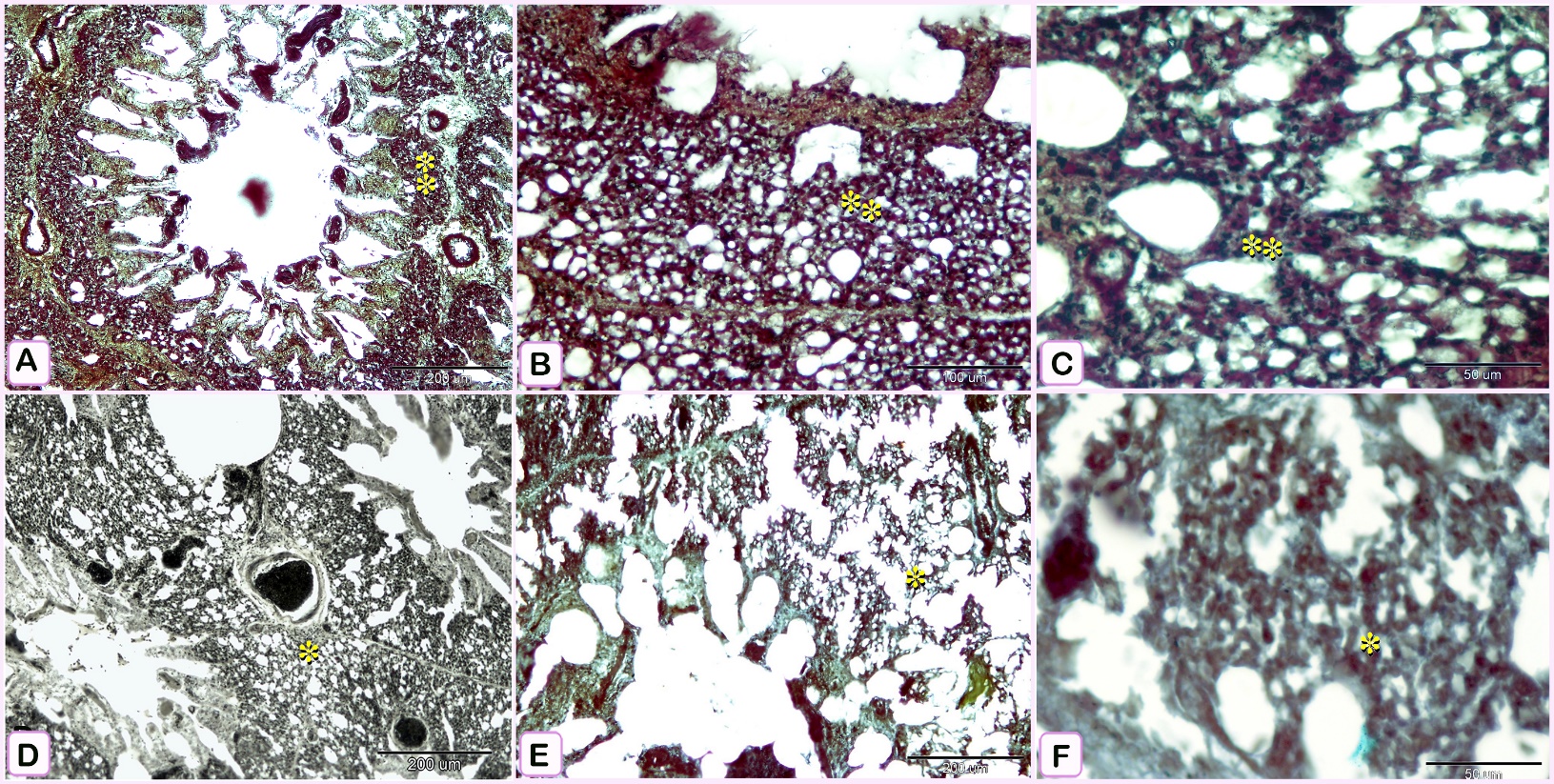


Fig. 5. paraffin sections of the lung were stained by mercury bromophenol blue for detection of protein deposition.

A-C: lung sample of the control group. Blue coloration indicated positive protein inclusions. Lung tissue including the pneumocytes had a high (**) affinity for mercury bromophenol blue that indicate cellular inclusions of protein nature that was uniformly distributed. D-F: lung sample of groups of burning maize was stained by mercury bromophenol blue. pneumocytes had a low affinity (*) for bromophenol blue that was indicated by a marked decrease in protein inclusions. considerable loss and uneven distribution of protein inclusions are detected.


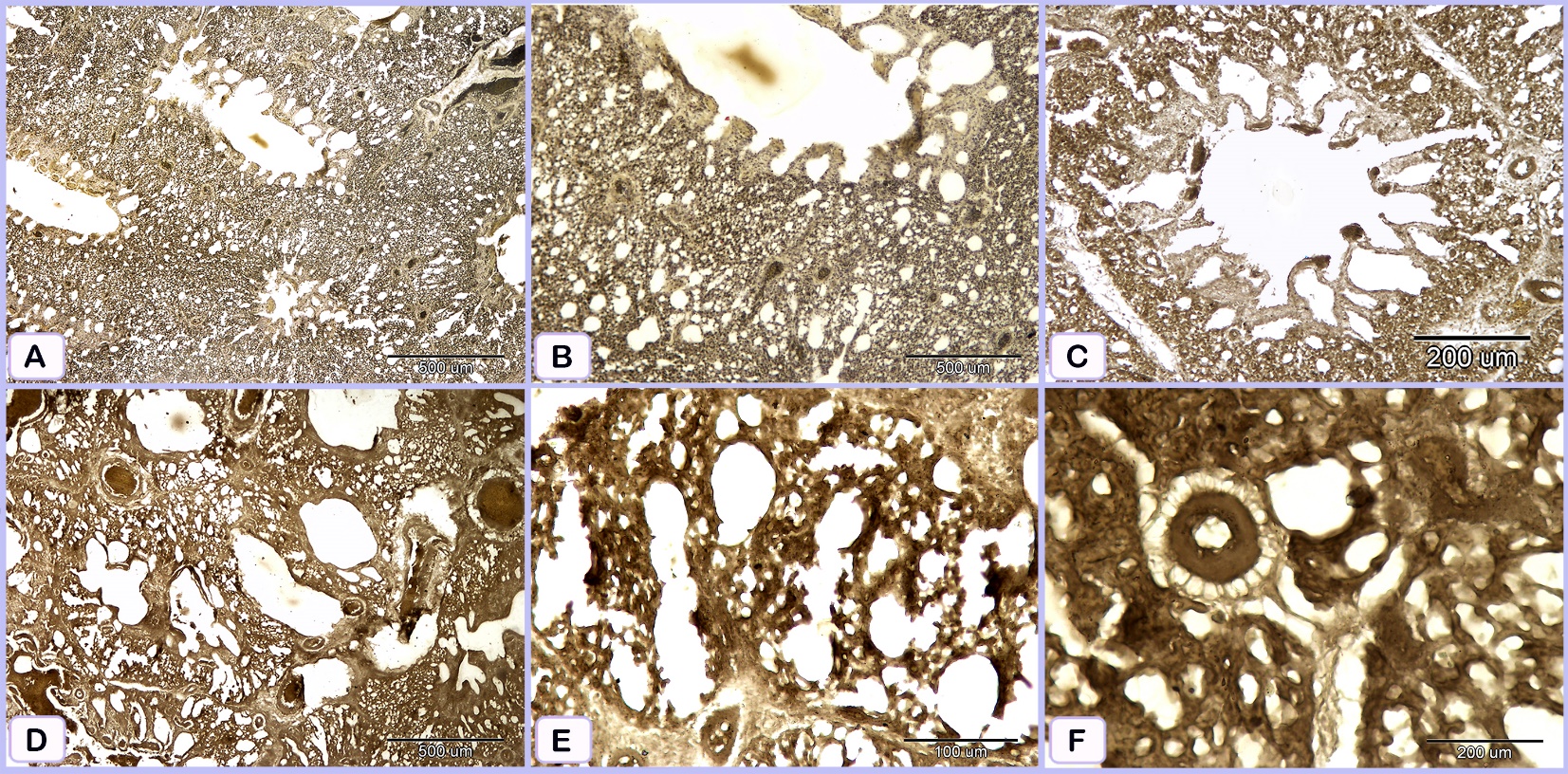


Fig. 6. paraffin sections of lung were stained by Gomori calcium method for detection of alkaline phosphatase activity.

A-C : lung sample of the control group. Lung tissue had a low affinity for alkaline phosphatase. D-F: lung sample of the groups of burning maize. lung tissue had a high affinity for alkaline phosphatase.


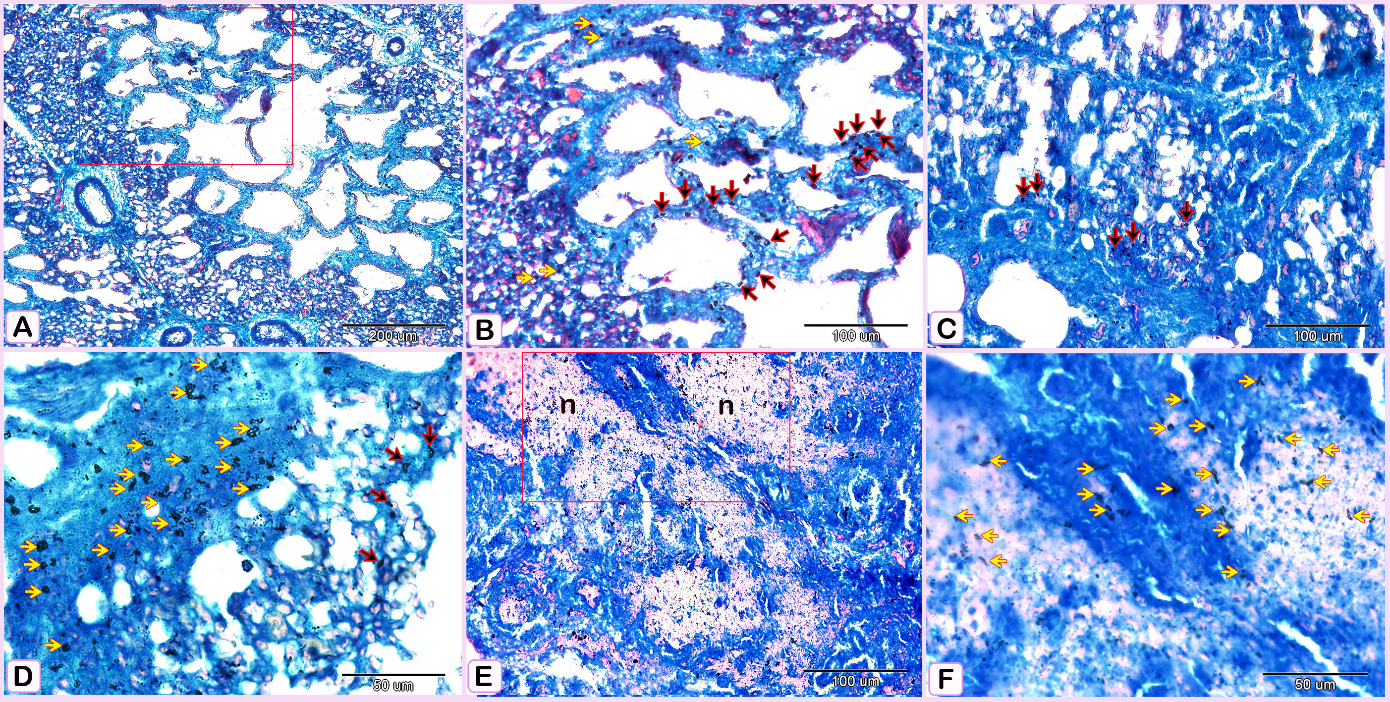


Fig. 7. paraffin sections of the lung were stained by pearls prussian blue for demonstration of

Hemosiderin pigments.

A, B: lung sample of the control group. Hemosiderin pigments inside the macrophage cells (black arrows) and few pigments withinthe interstitial tissue (yellow arrows).

C, D: lung sample of the groups of burning maize with a low number of macrophage with hemosiderin pigment (black arrows) and abundant pigment within the interstitial tissue (yellow arrows). E , F: necrotic areas (n) exhibited hemosiderin pigments ( yellow arrows).


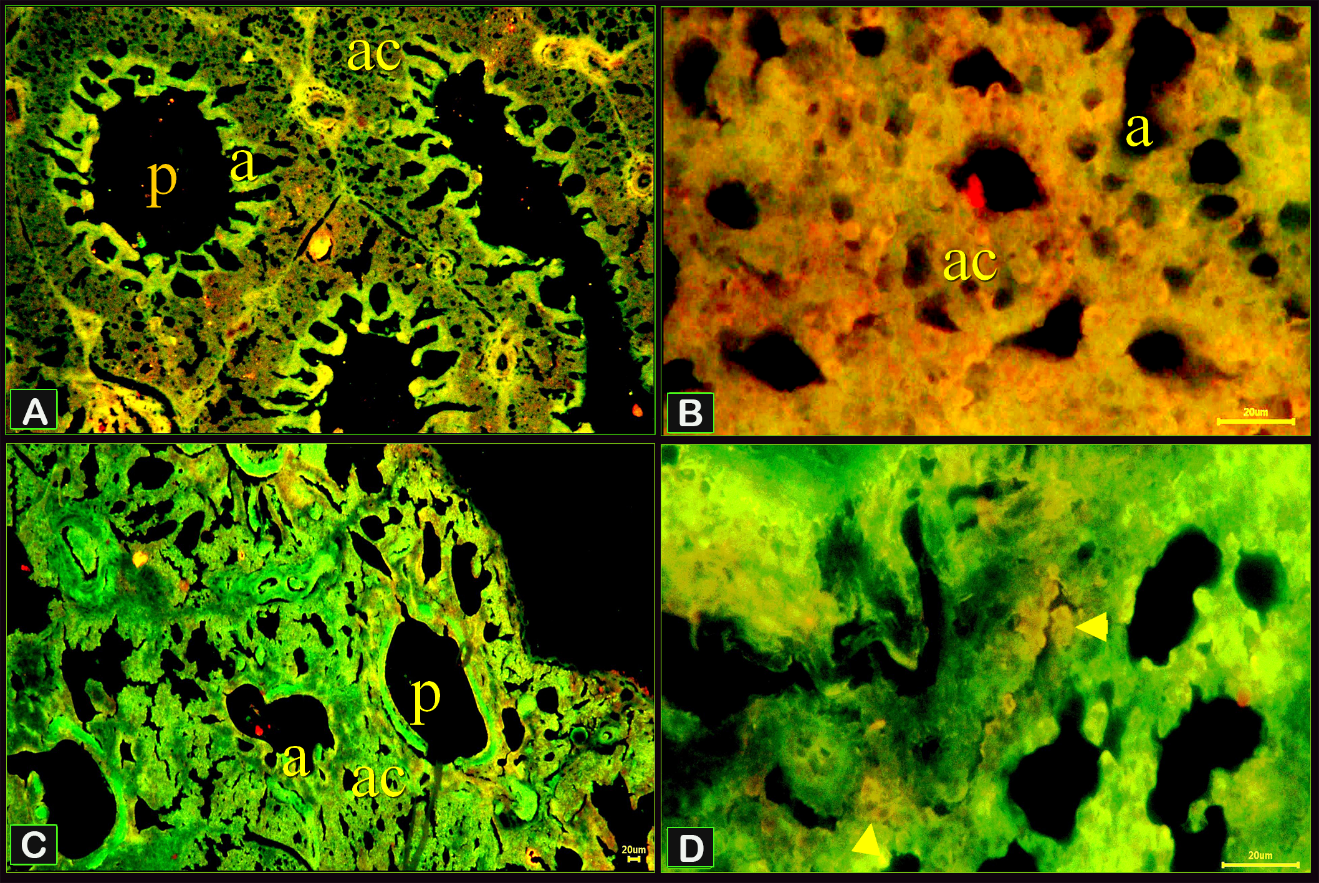


Fig. 8. paraffin sections of lung were stained by Acridine orange method for detection lysosomes activity

A, B: lung sample of the control group. Numerous activated lung macrophages that indicated by the yellow and red reaction. C, D: lung sample of the groups of burning maize. Less yellowish reaction (yellow arrowheads) indicated dismissing the lung macrophages activities. Note parabronchus(P), atria (a), air capillaries (ac).


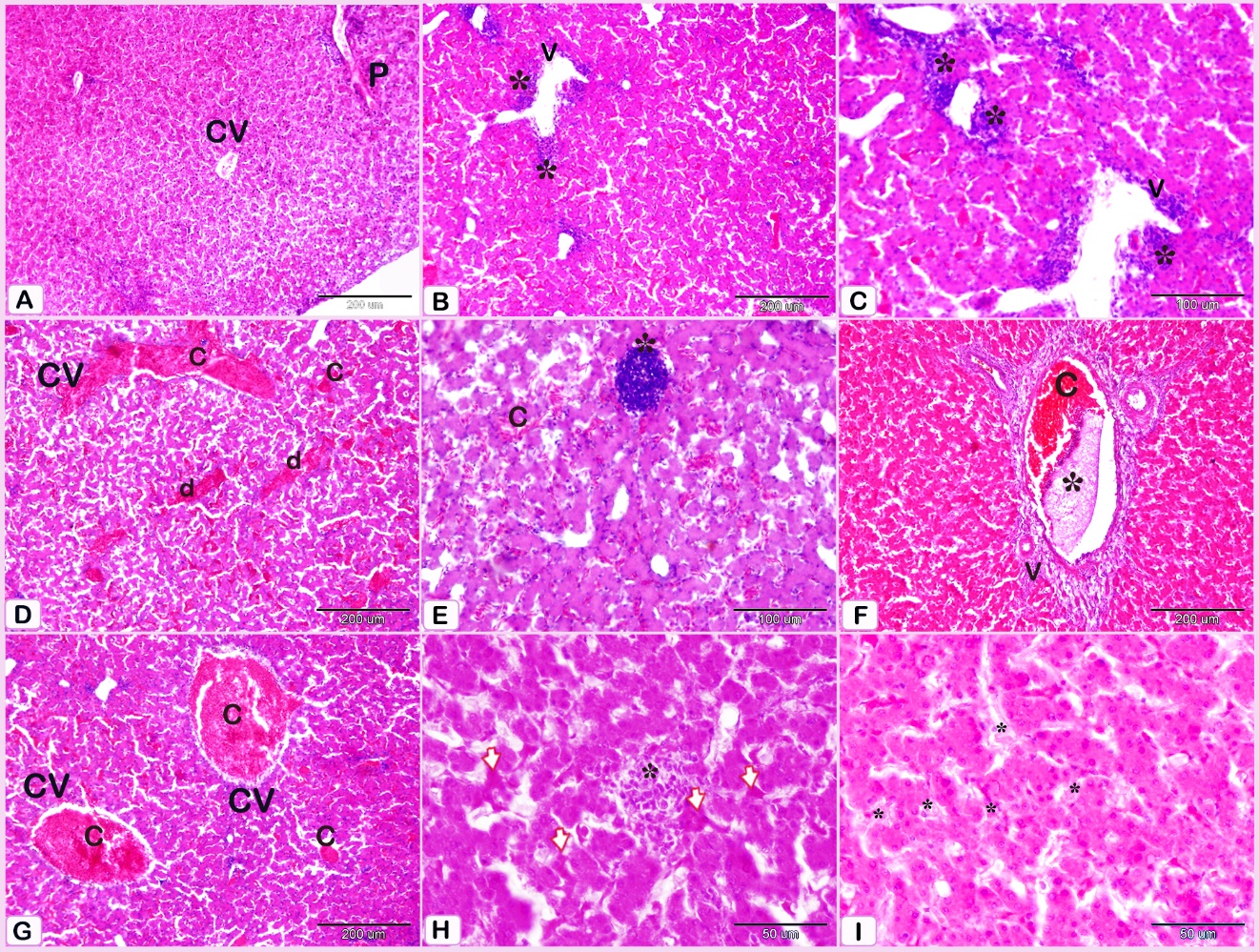


Fig 9: paraffin sections of liver were stained by Hematoxylin and Eosin (HE)

A: control sample of the liver showed normal hepatocytes and no leukocytic infiltration. Note central vein (cv) and portal area (P). B, C: vasculitis (v) and perivascular vascular lymphatic or leukocytic infiltration (*). D, G: congestion (C) of the central vein and the blood sinusoids. Dilation (d) and obstruction of the blood sinusoids F: embolic masses (asterisk) and vasculitis (V) of the blood vessels of the portal area, venous congestion (C), embolic masses (asterisk) in the vein (*). E, H: Nodular lymphatic infiltration (asterisk). Acidophilic bodies of the necrotic cells (arrows). I: embolic masses (asterisk) in the blood sinusoids.


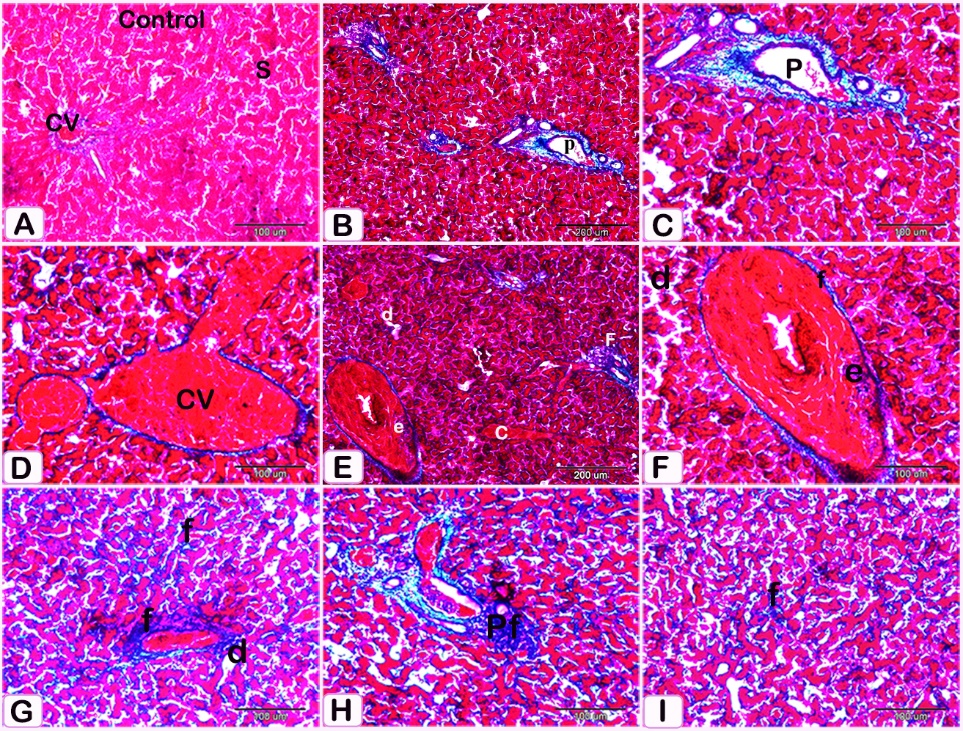


Fig 10: paraffin sections of liver were stained by Crossman’s trichrome for detection of fibrosis.

A, Control group showing central vein (CV) and liver sinusoids (S) without fibrosis. B-F samples from groups of burning maize showing in B and C: increase connective tissue in the portal areas (P) and perivascular connective tissue. Note portal veins (P). D: dilatation and congestion of the central vein (CV). E, F: central vein showed an embolus (e), fibrotic changes (f), dilatation (d) of the blood sinusoids. G-I: interstitial and perivascular fibrosis (f), dilatation (d) of the blood sinusoids.


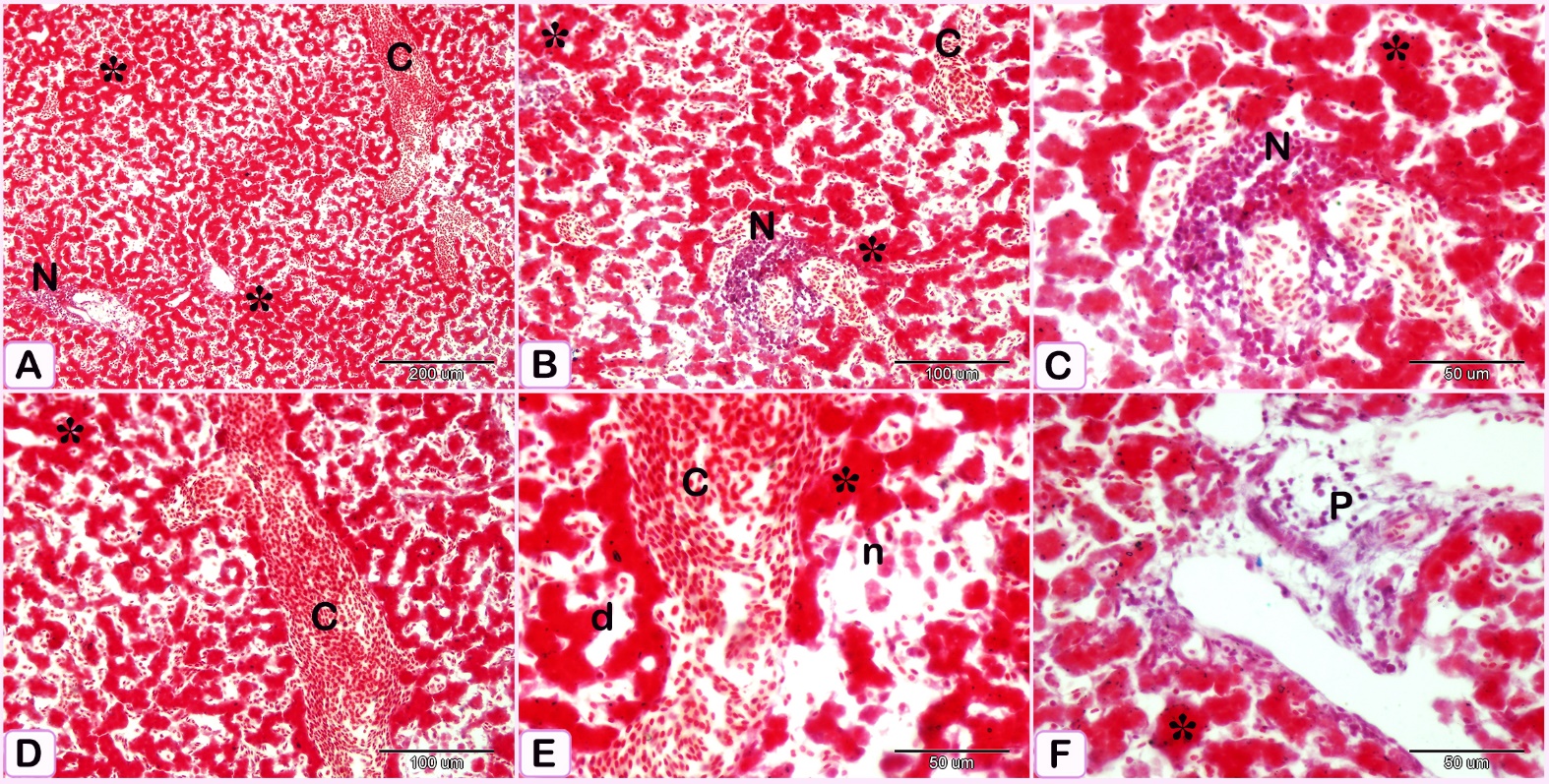


Fig 11: paraffin sections of liver samples from groups of burning maize were stained by Long zheil

Nielson stain showing the Lipofuscin pigment.

A-F: hepatocytes exhibited a strong positive reaction for Long zheil Nielson stain for Lipofuscin

Pigment. dilated blood sinusoids (d), (n) Necrosis of hepatocytes, lymphocytic infiltrations in the

area (*). Congestion of the central vein (C). Nodular lymphatic infiltration (N), lymphocytic

infiltration in (P )portal area.


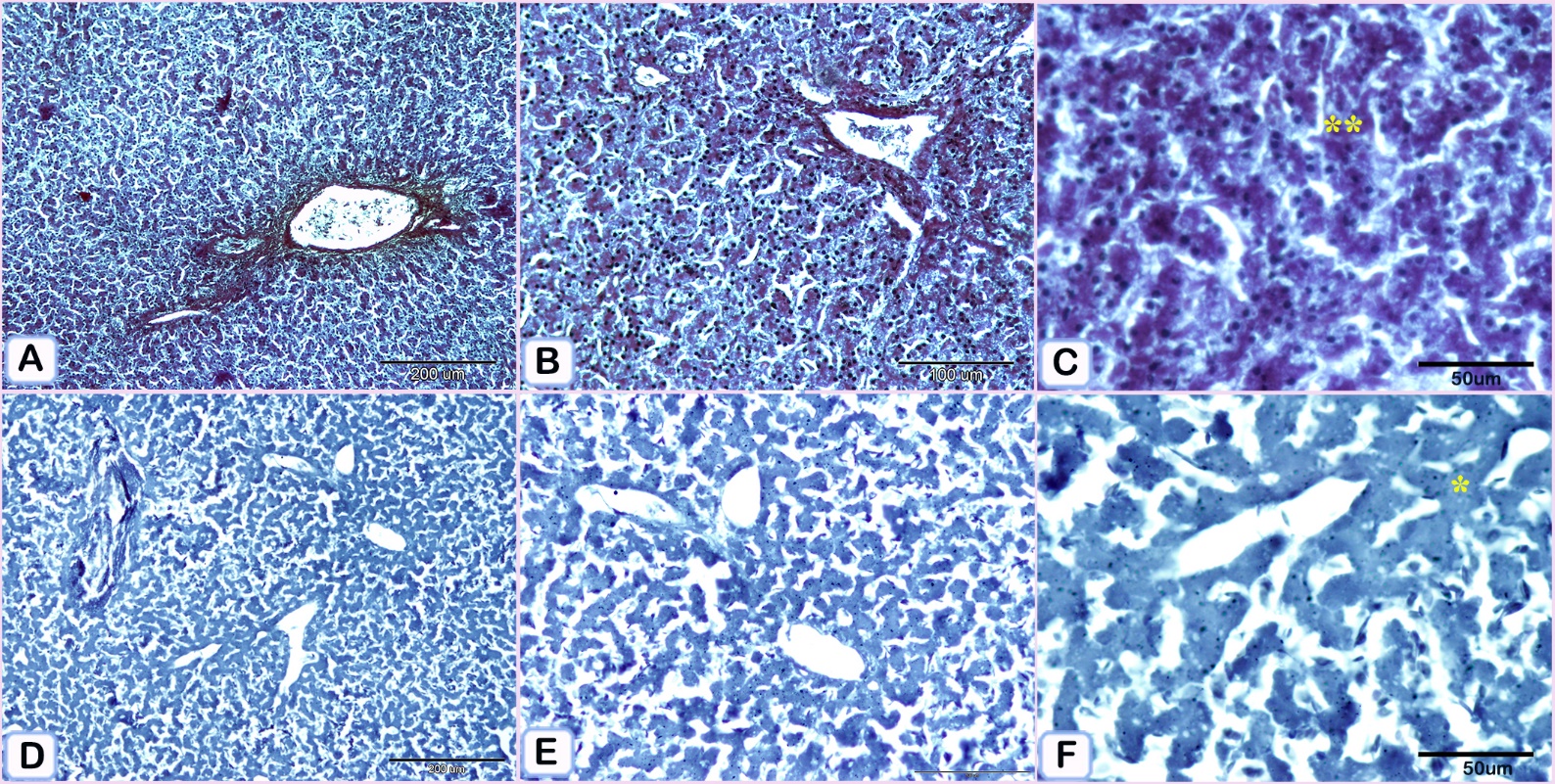


Fig12: paraffin sections of liver were stained by mercury bromophenol blue for detection of protein deposition.

A, B, C: liver sample of the control group. Blue coloration indicated positive protein inclusions. Hepatocytes had a high (**)affinity for mercury bromophenol blue that indicate cellular inclusions of protein nature that was uniformly distributed in the hepatocytes. D, E, F: liver sample from groups of burning maize showing the Hepatocytes had a low affinity (*) for bromophenol blue that was indicated by a marked decrease in protein inclusions of the hepatocytes (HP). considerable loss and uneven distribution of protein inclusions are detected.


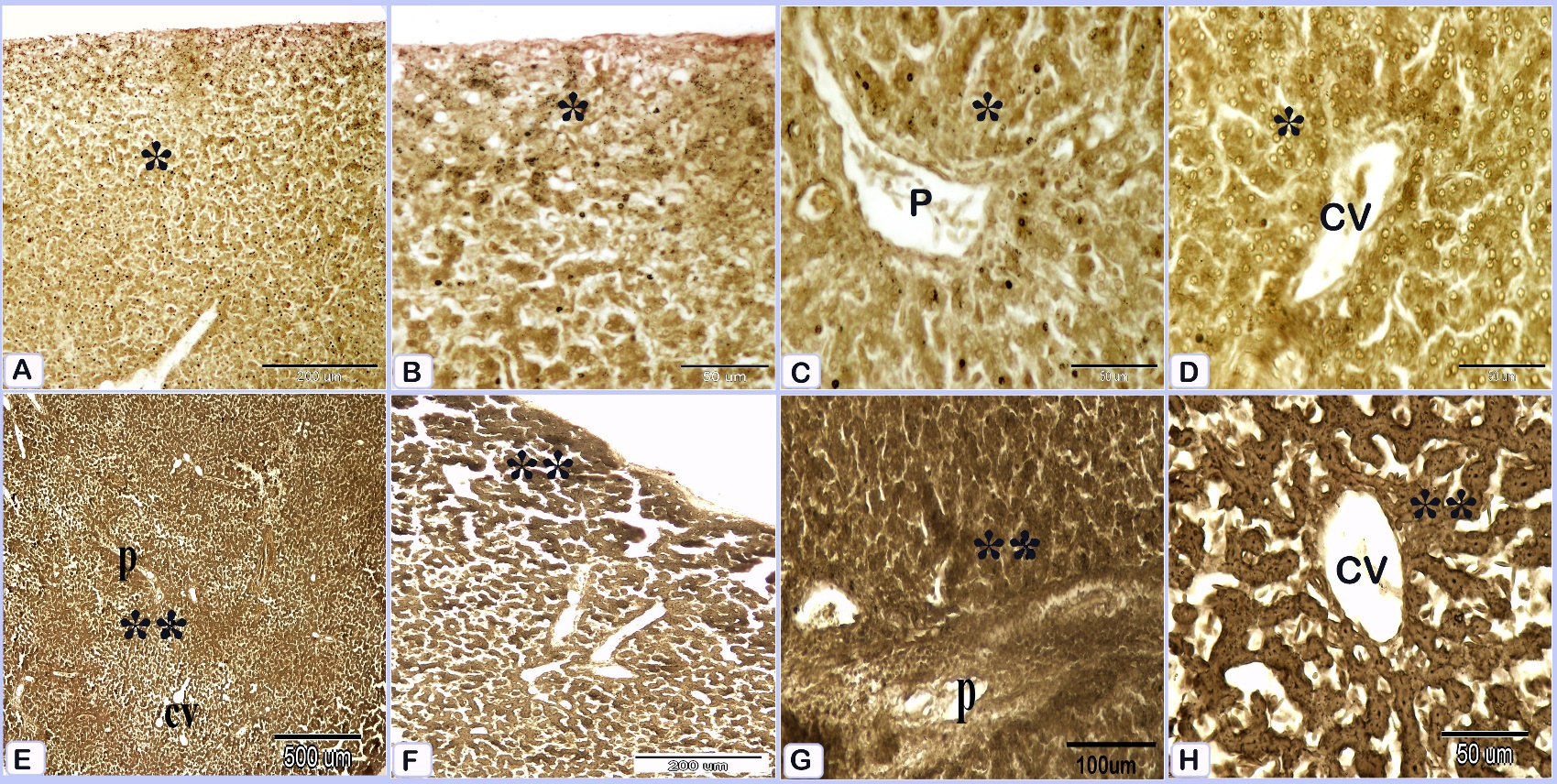


Fig 13: paraffin sections of liver were stained by Gomori calcium method for detection of alkaline phosphatase activity. Liver sample of the control group (A-D) and a Liver sample of the samples from groups of burning maize showing group (E-H).

A: Hepatocytes had low affinity for alkaline phosphatase. B, C: alkaline phosphatase activities of hepatocytes varied according to metabolic zonation of the liver the hepatocytes in the periportal zone (asterisk in Fig. (B)) had less alkaline phosphatase activities than hepatocytes in the Centrilobular zones (asterisk in Fig. (C)). D: the subcapsular hepatocytes (asterisk) had low affinity for alkaline phosphatase.

E: Hepatocytes (double asterisk) had a high affinity for alkaline phosphatase. Note: ce

ntral vein (CV), portal area (P). F:The subcapsular hepatocytes (double asterisk) had a high affinity for alkaline phosphatase. G: Hepatocytes in the periportal zone (double asterisk) had a high affinity for alkaline phosphatase. H: Hepatocytes Centrilobular zones (double asterisk) had a high affinity for alkaline phosphatase.


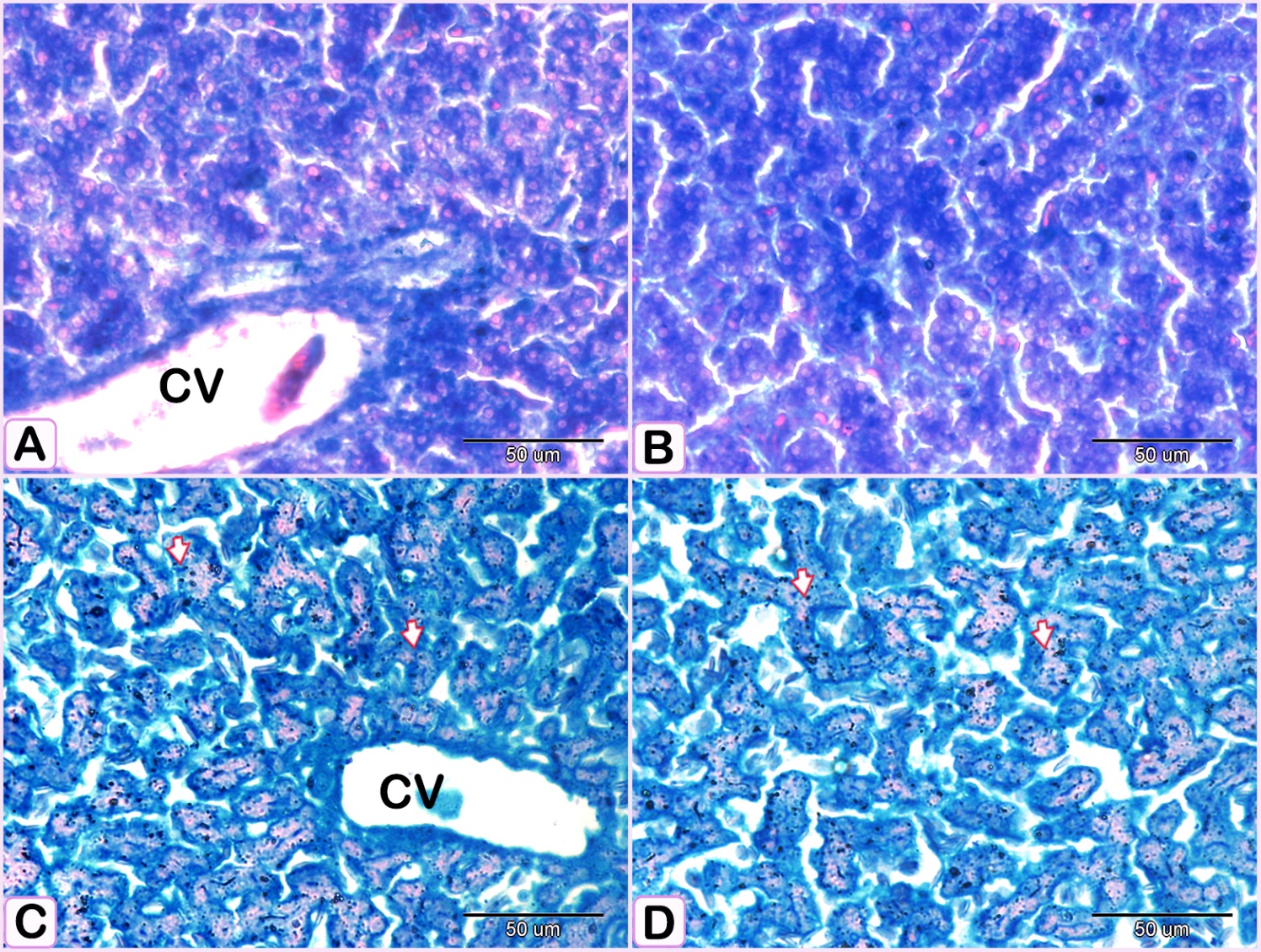


Fig. 14: paraffin sections of liver were stained by Pearls Prussian blue for demonstration of hemosiderin pigments

A, B: liver sample of the control group. Hepatocytes in the Centro-lobular zone free from hemosiderin pigments. C: hepatocytes in the Centro-lobular zone exhibited hemosiderin pigments (arrows). D: hepatocytes exhibited hemosiderin pigments (arrows). Note central vein (CV).


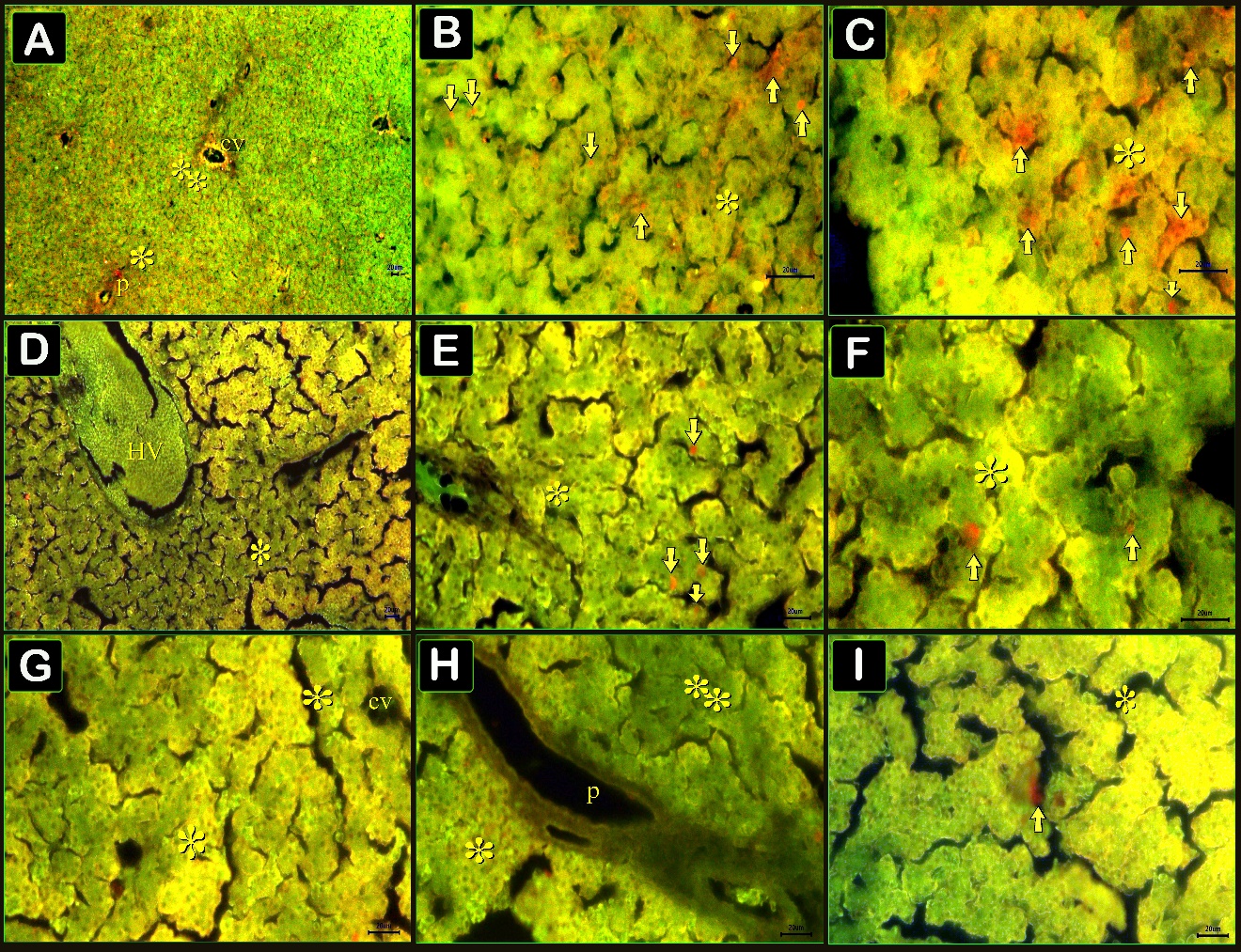


Fig. 15. paraffin sections of liver were stained by Acridine orange method for lysosomes.

Liver sample of the control group (A-C) and a Liver sample of the samples from groups of burning maize showing group (D-I).

A, B, C: liver sample of the control group. Note numerous activated kupffer cells indicated by the yellow and red reaction. A: Hepatocytes in the periportal zone (*) stained intense yellow compared with the weak yellow in the Centrilobular zones (**). Note portal area (P), central vein (CV). B, C: hepatocytes exhibited yellowish reaction (*) and numerous kupffer cells (arrows) stained red.

D-I: liver sample of the groups of burning maize. Less red reaction indicated dismissing the kupffer cells activities. yellow, orange-red reactions indicate lysosome activity.  D-F, I: hepatocytes exhibited intense yellowish reaction (*) and few kupffer cells (arrows) stained red. G: hepatocytes exhibited intense yellow in the Centrilobular zones (*). H: Hepatocytes in the periportal zone (*) stained weak yellow


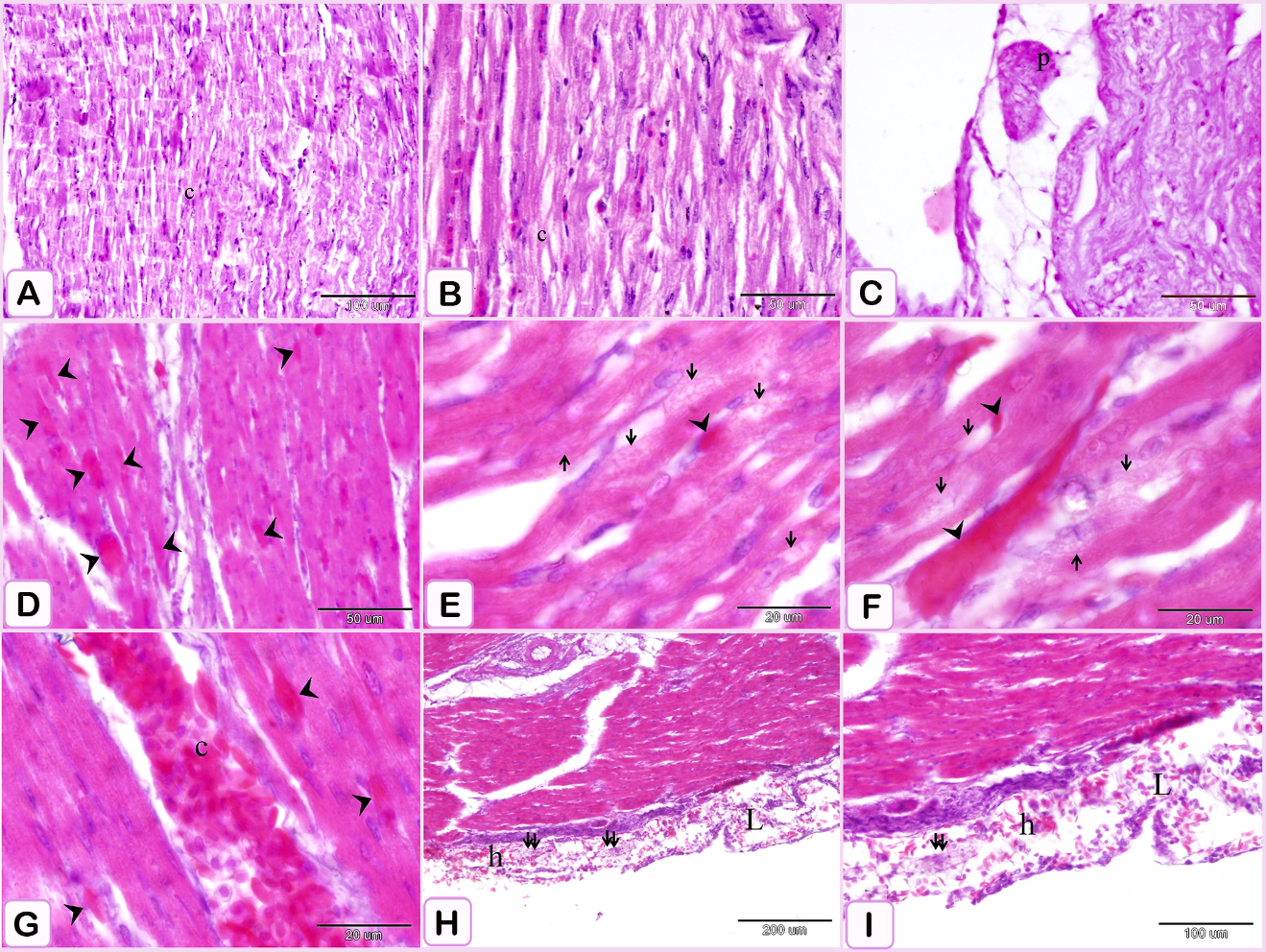


Fig 16: Photomicrograph of Hematoxylin and Eosin stained paraffin sections of the heart .

A-B: heart samples of the control group. Cardiomyocytes (c) arranged in a branched pattern. C: Purkinje cell fiber (p) had peripheral acidophilic cytoplasm and central faintly stained cytoplasm. D-F: heart sample from groups of burning maize showed intense acidophilic cytoplasm (arrowheads), cytoplasmic vacuolations (arrows) of the necrotic cardiomyocytes. G: Congested blood vessel (C). intense acidophilic cytoplasm of the necrotic cardiomyocytes (arrowheads). H, I: Subendocardial lymphoid infiltration (L), and hemorrhage (h), Degenerating Purkinjie cell fiber (double arrows).


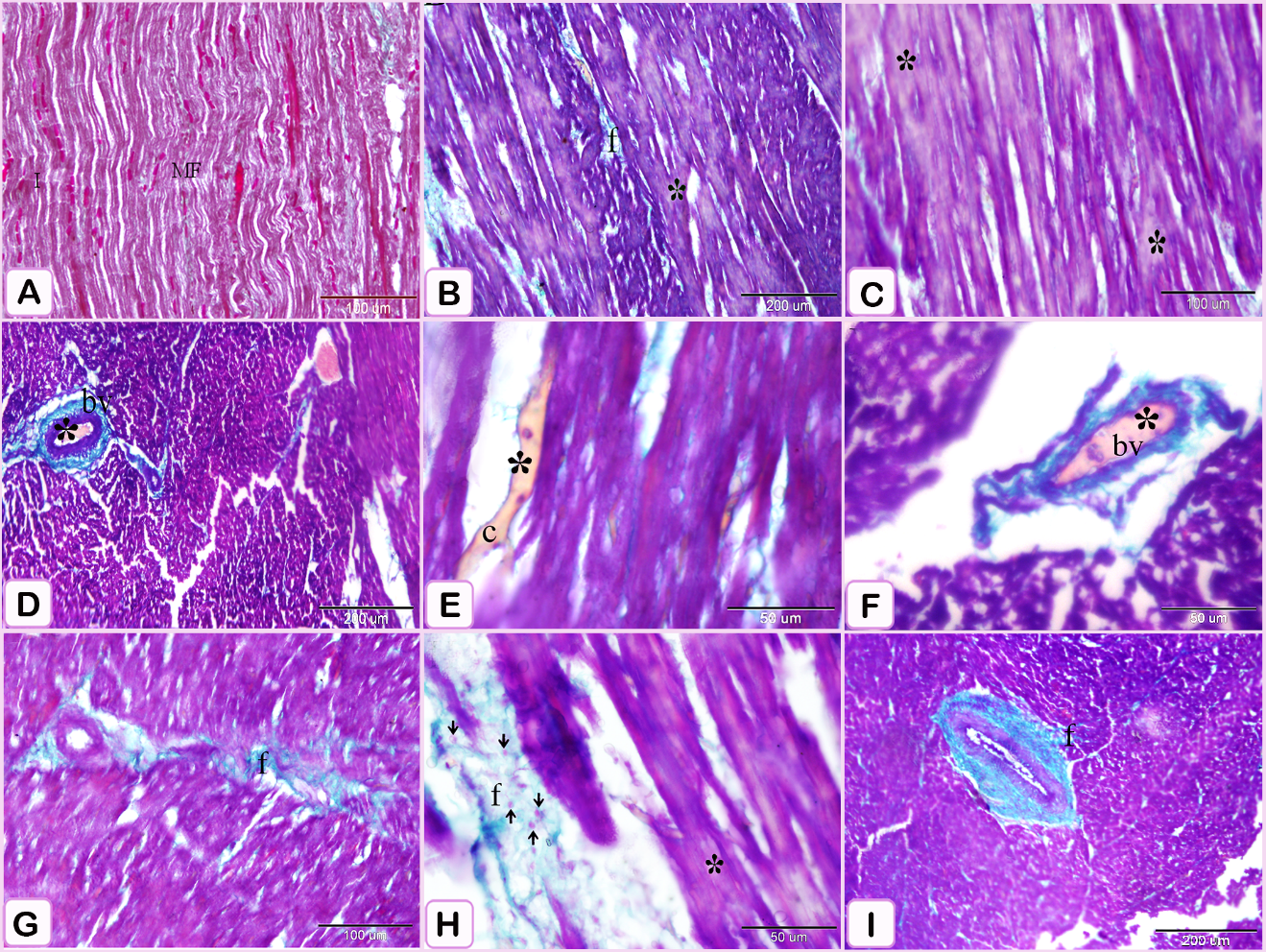


Fig. 17: Photomicrograph of Crossmon trichrome stained paraffin sections of the heart

A: Control samples. Note cardiomyocytes contained myofilaments (MF), intercalated disk (I). B, C, H: Degradation (asterisks) of the myofilaments was identified as Pale stained area exhibiting low affinity for acid fuchsin- orange G. Fibrotic changes in the interstitial tissue (f). inflammatory cells (arrows). D-F: Embolic events (asterisks) were detected in the blood vessels (bv) and capillaries (c). fibrotic changes (f). G, I: hypertrophy of the vascular wall (bv) and fibrotic changes (f) were observed


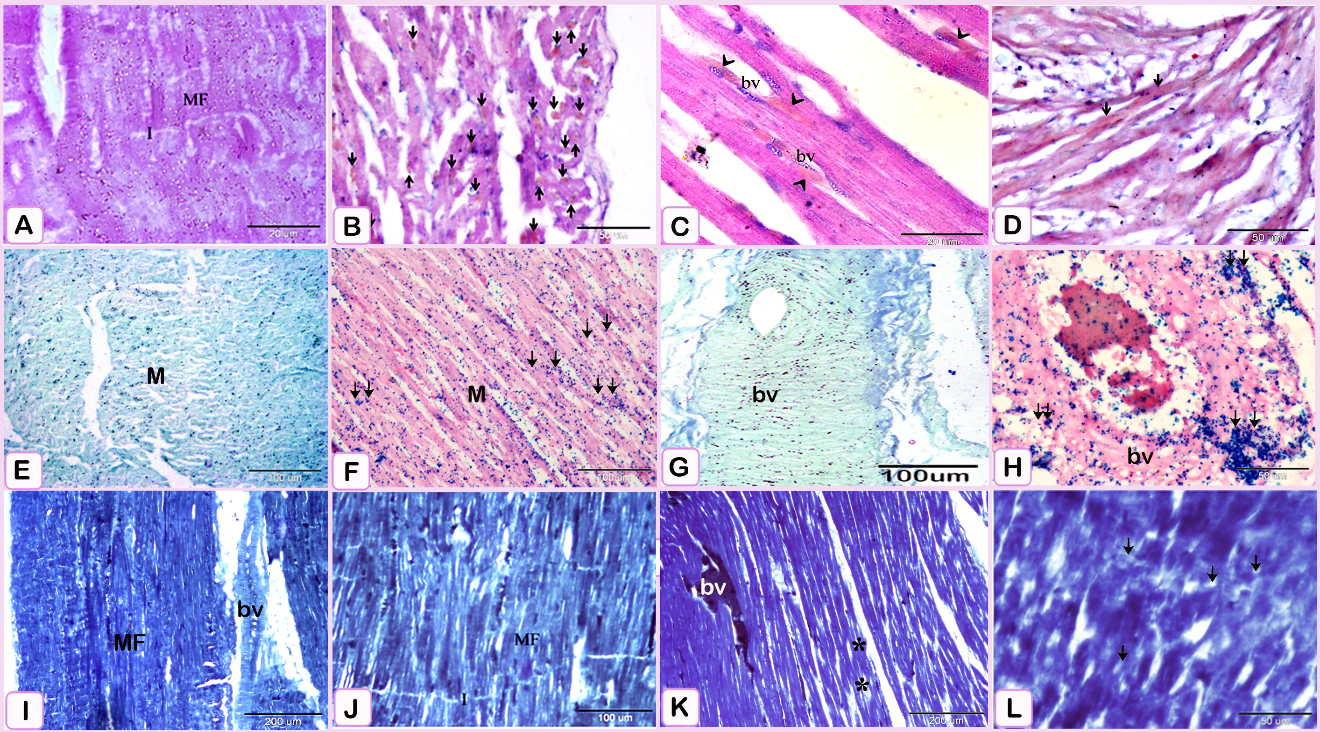


Fig. 18: Photomicrograph of Long zheil Nielson (A-D), Prussian blue (E-H), Mercury bromophenol blue (I-L) stained paraffin sections of the heart.

A, E, G, I, J, M: control samples. Note cardiomyocytes (M) contained myofilaments (MF), intercalated disk (I), blood vessel (bv).

B: Lipofuscin pigments (arrows) were detected using Long zheil Nielson. C: Lipofuscin pigments (arrowheads) were detected in the blood vessels (bv). D: Lipofuscin pigments (arrows) distributed in the whole cardiomyocytes. F, H: hemosiderin pigments (arrows) were detected in the cardiomyocytes(M) and in blood vessels (bv)(arrows). K-L: Degradation of the myofilaments appearing as dark homogenous areas(arrows). Areas of the cytoplasm of the cardiomyocytes exhibited complete destruction myofilaments (asterisks). Congestion of blood vessel (bv).


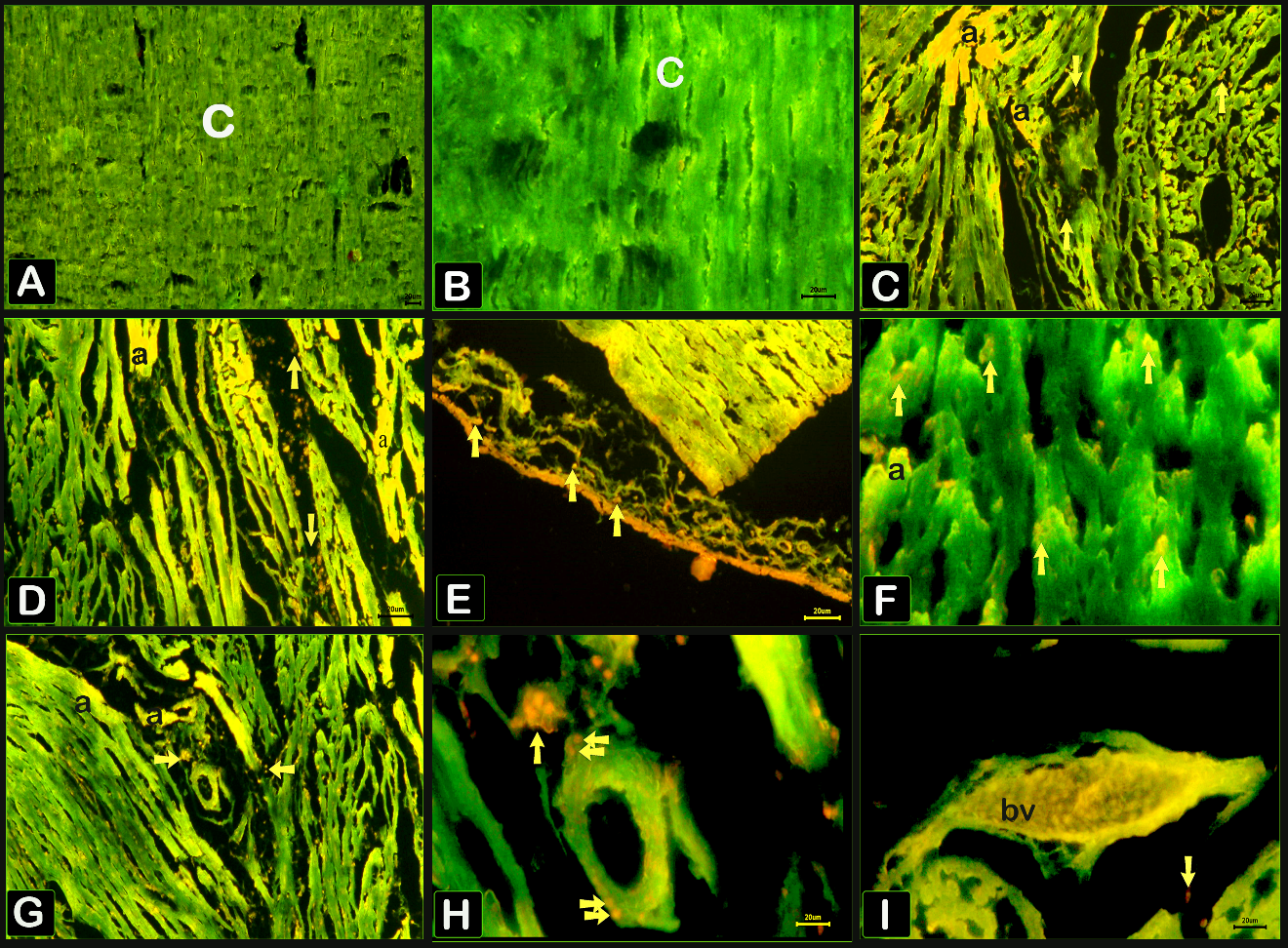


Fig 19: Fluorescent Photomicrograph of Acridine orange stained paraffin sections of the heart

A, B: control samples of the heart showed cardiomyocytes (C) stained green. C, D, F: interstitial inflammatory cells exhibited a yellow color that indicating lysosomes. Note lysosome-rich cardiomyocytes (a) stained yellow indicating apoptosis. E: endocarditis was identified by inflammatory cells rich in lysosomes (arrows). G, H: endomysial connective tissue was infiltrated by lysosome-rich inflammatory cells (arrows). Note inflammatory cells (double arrows) migrate through the wall of the blood vessels. I: lysosome-rich inflammatory cells (arrows) around the congested blood vessel (bv).


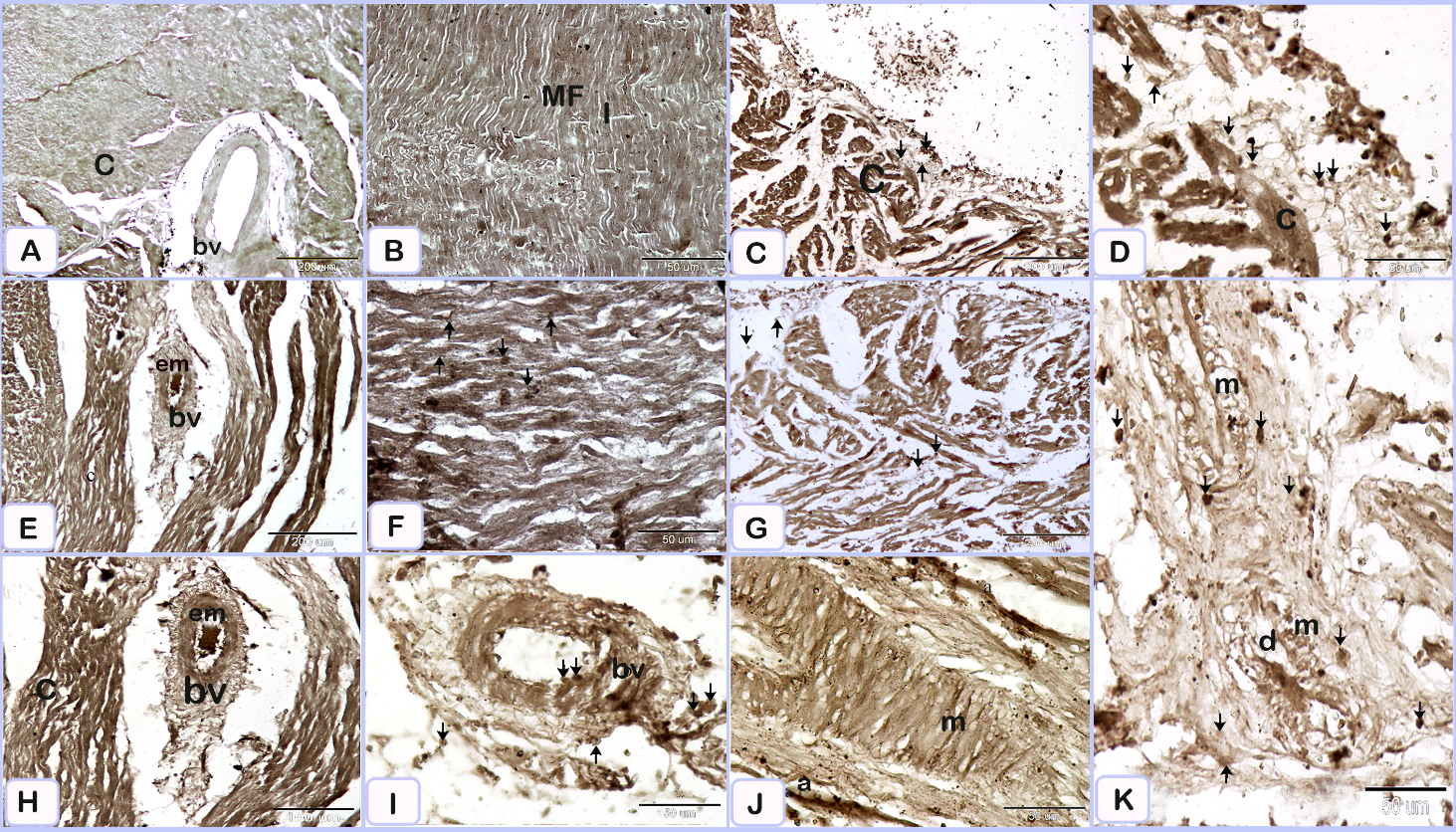


Fig. 20: Photomicrograph of paraffin sections of heart stained with Gomori calcium method for detection of alkaline phosphatase activity.

A, B: control samples of the heart. Note cardiomyocytes (C), blood vessel (bv), myofilaments (MF), intercalated disk (I).C, D: cardiomyocytes (C) exhibited high affinity for alkaline phosphatase. Infiltration of the inflammatory cells (arrows) in the subendocardium and between cardiomyocytes. E, H: cardiomyocytes (C) exhibited high affinity for alkaline phosphatase. Embolic change (em) was detected in the blood vessel (bv). Note muscular tunic of the blood vessel exhibited high affinity for alkaline phosphatase. F: cardiomyocytes (C) exhibited high affinity for alkaline phosphatase. Infiltration of the inflammatory cells (arrows) between cardiomyocytes. I, J: inflammatory cells (arrows) exhibited high affinity for alkaline phosphatase in the lumen of the blood vessel (bv) and perivascular tissue. G: a muscular tunic of the blood vessel exhibited high affinity for alkaline phosphatase. Note atrophic cardiomyocytes (a) exhibited high affinity for alkaline phosphatase. K: vascular wall underwent degeneration (d) of the endothelial cells and atrophied muscular tunic (m). note inflammatory cells (arrows) penetrating the wall of the blood vessel (bv).

Discussion

ROS are associated with inflammation and induce molecular changes through oxidative damage to proteins, lipids, and DNA [[63](https://mail.google.com/mail/u/0/#m_-3451482287856491428__ENREF_63)]. They also lead to necrosis and apoptosis [[64](https://mail.google.com/mail/u/0/#m_-3451482287856491428__ENREF_64)]. Decreased protein occurred in the lung associated with MSB which could indicate functional impairment related to a reduction in protein necessary for pulmonary surfactant and impairment of the synthesis of proteins such as A and clotting factors due to PM exposure [[65](https://mail.google.com/mail/u/0/#m_-3451482287856491428__ENREF_65)]. PM is involved in protein oxidation in the liver, and the subsequent activation of protein degradation pathways, including the ubiquitin-dependent and independent autophagy pathways, varies according to the type of the PM exposure [[66](https://mail.google.com/mail/u/0/#m_-3451482287856491428__ENREF_66)].Increased ALP activity in the lung, liver, and heart following chronic MSB exposure was identified and was associated with necrotic changes and tissue destruction. ALP is a plasma membrane-bound glycoprotein that hydrolyzes phosphate monoesters [[67](https://mail.google.com/mail/u/0/#m_-3451482287856491428__ENREF_67)]. Tissue destruction may result in ALP elevation in interstitial fluid and the bloodstream [[68](https://mail.google.com/mail/u/0/#m_-3451482287856491428__ENREF_68)]. Increased[[dm1]](https://mail.google.com/mail/u/0/#m_-3451482287856491428__msocom_1)  ALP activity due to tissue damage was noted in in MSB exposed broilers. The smaller particle size (PM2.5) had a stronger effect as it was deposited deeper in the lung tissue [[69](https://mail.google.com/mail/u/0/#m_-3451482287856491428__ENREF_69)]. The WHO assessed PM exposure to be associated with approximately 16% of lung cancer deaths, 11% of COPD deaths, and more than 20% of ischemic heart disease and stroke. However, although the exact mechanism for these health complications is still unclear, particle size, shape, number, and chemical composition are clearly important [[70](https://mail.google.com/mail/u/0/#m_-3451482287856491428__ENREF_70)]. PM_2.5_ could pass through the respiratory system, reach the blood stream, and be deposited along the endothelial walls and within several organs thus exerting severe damage throughout the body. A longer exposure to penetrable PM_2.5_ in the lung could be associated with systemic inflammation and oxidative stress [[71](https://mail.google.com/mail/u/0/#m_-3451482287856491428__ENREF_71)]. Inhalation of PM_2.5_ could be a significant stress factor inducing chronic liver disease (causing liver cirrhosis with the accumulation of extracellular collagen), pulmonary, and cardiovascular diseases [[72](https://mail.google.com/mail/u/0/#m_-3451482287856491428__ENREF_72)]. As a consequence, pathological changes including chronic atherosclerosis and angina, myocardial infarction, and cardiac arrest could be triggered with the possibility of death Carcinogenic events may occur as a result of the generation of ROS and various metabolic dysfunctions [[73](https://mail.google.com/mail/u/0/#m_-3451482287856491428__ENREF_73)].

Exposure to PM was associated with systemic pro-inflammatory mediators production which had a potential role in ischemic cardiovascular and atherosclerotic induction ([Fornasini and Bravo, 2015](#_ENREF_18)). Inhalation of PM was linked to infiltration of the lung neutrophils and the pro-inflammatory cytokines (tumour necrosis factor-α (TNF-α) and interleukin-1β (IL-1β)) and impairment of the phagocytic activity of the alveolar macrophages ([Lundborg et al., 2006](#_ENREF_46); [Aalapati et al., 2014](#_ENREF_1)).

Ultrafine particles exposure may also be related to diminishing the anti-inflammatory effect of plasma high-density lipoprotein and increasing the systemic oxidative stress, which alters the hepatic function indicated by elevation of hepatic malondialdehyde levels and upregulation of Nrf2-regulated antioxidant genes ([Araujo et al., 2008](#_ENREF_4)). Exposure to PM generates production of high levels of ROS in respiratory tract including A549 cells ([Tang et al., 2015](#_ENREF_78)), alveolar macrophages ([Lundborg et al., 2006](#_ENREF_46)), normal bronchial epithelial (BEAS-2B) cells ([Kim et al., 2011](#_ENREF_37)).

Generation of ROS during exposure to PM ([Li et al., 2003](#_ENREF_42)) had been linked to mitochondrial damage ([Li et al., 2003](#_ENREF_42)).

**4.3 Histopathological evidence of cellular damage due to MSB**

MSB results in the release of CO and fine PM which result in severe pneumonia, hepatitis, and carditis that may be identified by nodular and diffuse forms of mononuclear inflammatory cell infiltrations. Exposure to PM was responsible for thrombogenic events as well as hypertrophic, metaplastic, and necrotic changes. CO directly destroys lung tissue prior to the formation of the COHb which changes the capillaries permeability resulting in the leakage of macromolecules out of the vasculature and hypoxia ([Huang, Pan et al. 2017](#_ENREF_21)). Metaplastic conversion occurs by the transformation of pulmonary fibrous tissue into cartilaginous nodules which may be considered a premalignant stage of cancer ([Gazdar and Brambilla 2010](#_ENREF_14)). Fibrocartilaginous metaplasia may occur as a result of unbalanced regulation between growth factors, particularly TGF beta (fibroblast cell lineage) and Sox9 (chondrogenic lineage), and could be associated with the expression of types I and II collagen, S-100 protein, and chondroitin sulfate ([Lorda-Diez, Montero et al. 2009](#_ENREF_32)). Epithelial-mesenchymal transition (EMT) as a response to PM2.5 metaplasia could occur due to excessive ROS, and certain components of PM_2.5_, could activate singling pathways that alter cytoskeletal origination ([Xu, Ding et al. 2019](#_ENREF_61)). In the current study, MSB exposed tissue exhibited a low affinity for elastic stains indicating the loss of elasticity required for respiratoration. Repair of lung elasticity following interstitial pulmonary damage requires further studies to explore the ability of pulmonary fibroblasts to secrete elastic fibers.

Fibrosis was estimated to be 38.8%, 23.5%, and 15.4% in the lung, heart, and liver, respectively. Zhang attributed extensive lung damage to the direct deposition of PM_2.5_ in the lung tissue rather than deposition via systemic circulation ([Zhang, Gao et al. 2016](#_ENREF_63)). [Yoo et al., 2019](#_ENREF_97) found a 29.6% incidence of lung cancer in the fibrotic areas and a 44.4% incidence of fibrosis. Accordingly, we suggest that the PM_2.5_ received by the MSB group would have subjected them to a high risk for cancer development in the areas with pulmonary fibrotic changes. The fibrotic changes in the heart and the liver were limited and likely insufficient to induce oncogenesis. Up to 80 – 90% of cases with liver fibrosis develop liver cancer ([O'Rourke, Sagar et al. 2018](#_ENREF_40)), while the progression of fibrosis to cancer in the heart is uncommon ([Parichatikanond, Luangmonkong et al. 2020](#_ENREF_45)).

 Fibrosis can promote the initiation, progression, and metastasis of cancer. Fibrotic changes depend on activation of fibroblasts and stem cells, both of which have phenotypic features of cancer cells. Cancer-associated fibrosis has a significant influence on the tumor microenvironment. Lung cancer-associated with interstitial lung diseases (ILDs) has been linked to cigarette smoking which characterized by a rapid proliferation of fibroblasts and pulmonary fibrosis ([Königshoff 2011](#_ENREF_28)).. The epithelial cells fail to regenerate which in turn activates growth factors leading to fibroblast accumulation, rapid epithelial proliferation, hyperplasia, and metaplasia. Epithelial activation acts as a nidus for lung cancer development ([Königshoff 2011](#_ENREF_28)). PM_2.5_ exposure is related to EMT and induction of cancer stem cells (CSC). Acute and chronic PM_2.5_ exposure induces cell migration and invasion, diminishes mRNA expression of epithelial markers, and increases mRNA expression of mesenchymal markers indicating fibrosis. Chronic PM_2.5_ exposure increases cell-surface markers (CD44, ABCG2), self-renewal genes (SOX2 and OCT4), side population cells, and neoplastic activity. Chronic PM_2.5_ exposure drives cells toward malignant behavior indicated by EMT and CSC properties ([Wei, Liang et al. 2017](#_ENREF_58)). In the current study, exposure to PM_2.5_ led to pulmonary fibrosis which is associated with epithelial degeneration and metaplastic changes of interstitial and epithelial tissue suggesting that MSB exposure may act as a potent risk factor leading to lung cancer.

Myocardial and vascular degeneration are associated with the infiltration of inflammatory cells causing carditis. PM2.5 exposure is related to atherosclerosis, cardiovascular mortality, ischemic heart disease, and ventricular arrhythmias ([Fornasini and Bravo 2015](#_ENREF_11)). the effects of particles on cardiac muscle occurs through particles reaching the bloodstream and spreading directly to the heart and through the oxidative stress and inflammatory reactions ([Huang, Choi et al. 2019](#_ENREF_22)). PM2.5 exposure is responsible for inducing thrombogenic events which may be related to the loss of homeostasis in the pro-thrombotic/pro-coagulative state. Platelet activation, oxidative stress, the interaction of interleukin-6 with other tissue factors, as well as the effect of circulating microvesicles and epigenetic changes ([Robertson and Miller 2018](#_ENREF_50)).

Increased ALP activity in the lung, liver, and heart following chronic MSB exposure was associated with necrotic changes and tissue destruction. Tissue destruction may result in ALP elevation in interstitial fluid and the bloodstream ([Reynolds 2016](#_ENREF_49)). The WHO assessed PM exposure to be associated with approximately 16% of lung cancer deaths, and more than 20% of ischemic heart disease and stroke. However, although the exact mechanism for these health complications is still unclear, particle size, shape, number, and chemical composition are clearly important ([Liu, Pereira et al. 2015](#_ENREF_31)). PM_2.5_ could pass through the respiratory system, reach the blood stream, and be deposited along the endothelial walls and within several organs thus exerting severe damage throughout the body.

**5. Conclusion**

Sever environmental pollution (higher of Co and PM_2.5_ levels) were reported from the surrounding environment during MSB season. Furthermore, adverse health effect in form of pneumonia, carditis and hepatitis were observed following MSB exposure.  The lungs were the main organ affected with pulmonary fibrosis (38.8%) resulting in airway obstruction and diminished respiratory performance. The fibrotic and metaplastic lungs were at great risk for subsequently developing cancer. Embolic events in the vascular tissue of the lung, heart and liver indicted systemic embolism and cardiovascular disease risk. The importance of air quality monitoring is crucial for assessing regional emissions of excess leftover straw burning. This study found that exposure to agriculture waste burning is a potential hazard to human health and may lead to the development of cancer and CVD. Repeated annual exposure to MSB may increase health risks and lead to the development of cancer, as well as liver and heart failure. Further research comprising long-term monitoring of annual exposure and biomarker levels for cancer of different body tissues should be compulsory to estimate the degree of risk for humans.
